# Re-wiring of energy metabolism promotes viability during hyperreplication stress in *E*. *coli*

**DOI:** 10.1371/journal.pgen.1006590

**Published:** 2017-01-27

**Authors:** Godefroid Charbon, Christopher Campion, Siu Hung Joshua Chan, Louise Bjørn, Allan Weimann, Luís Cláudio Nascimento da Silva, Peter Ruhdal Jensen, Anders Løbner-Olesen

**Affiliations:** 1 Dept. of Biology, Section for Functional Genomics and Center for Bacterial Stress Response and Persistence, University of Copenhagen, Copenhagen, Denmark; 2 National Food Institute, Microbial Biotechnology and Biorefining, Technical University of Denmark, Lyngby, Denmark; 3 Laboratory of Clinical Pharmacology, Rigshospitalet, Section Q7642, Copenhagen Denmark and Department of Clinical Pharmacology, Bispebjerg Frederiksberg Hospitals, Copenhagen Denmark; Massachusetts Institute of Technology, UNITED STATES

## Abstract

Chromosome replication in *Escherichia coli* is initiated by DnaA. DnaA binds ATP which is essential for formation of a DnaA-*oriC* nucleoprotein complex that promotes strand opening, helicase loading and replisome assembly. Following initiation, DnaA^ATP^ is converted to DnaA^ADP^ primarily by the Regulatory Inactivation of DnaA process (RIDA). In RIDA deficient cells, DnaA^ATP^ accumulates leading to uncontrolled initiation of replication and cell death by accumulation of DNA strand breaks. Mutations that suppress RIDA deficiency either dampen overinitiation or permit growth despite overinitiation. We characterize mutations of the last group that have in common that distinct metabolic routes are rewired resulting in the redirection of electron flow towards the cytochrome bd-1. We propose a model where cytochrome bd-1 lowers the formation of reactive oxygen species and hence oxidative damage to the DNA in general. This increases the processivity of replication forks generated by overinitiation to a level that sustains viability.

## Author Summary

In most bacteria chromosome replication is initiated by the DnaA protein. In *Escherichia coli*, DnaA binds ATP and ADP with similar affinity but only the ATP bound form is active. An increased level of DnaA^ATP^ causes overinitiation and cell death by accumulation of DNA strand breaks. These strand breaks often result from forks encountering gapped DNA formed during repair of oxidative damage. We provide evidence that cell death in overinitiating cells can be prevented by rewiring the metabolism to favor the micro-aerobic respiratory chain with the cytochrome bd-1 as terminal oxidase. Cytochrome bd-1 is found in aerobic as well as anaerobic bacteria. Its role is to reduce O_2_ in micro-aerobic conditions and work as an electron sink to prevent the formation of reactive oxygen species. Our results suggest that bacteria can cope with replication stress by increasing respiration through cytochrome bd-1 to reduce the formation of reactive oxygen species, and hence oxidative damage to a level that does not interfere with replication fork progression.

## Introduction

Initiation of chromosome replication from the unique replication origin of *E*. *coli oriC*, is tightly controlled and happens once and only once per cell cycle [1, 2]. Chromosome replication is initiated by the DnaA initiator protein. DnaA is an AAA+ ATPase that exists in an ATP bound form and an ADP bound form [3]. DnaA associated with either ATP or ADP binds a set of strong recognition sites in *oriC* throughout the cell cycle [4] to form the origin recognition complex (ORC;[5]). Upon initiation the DnaA protein associated with ATP forms the orisome by binding to numerous additional sites in *oriC*. This displaces Fis (Factor for Inversion Stimulation), a protein that binds *oriC* for most of the cell cycle. With Fis gone, the IHF (Integration Host Factor) protein can bind *oriC*, which ultimately leads to duplex opening [6, 7], helicase loading and assembly of two replisomes [8].

The level of DnaA^ATP^ fluctuates during the cell cycle and is high at the time of initiation [9]. Following initiation, DnaA^ATP^ is converted to DnaA^ADP^ by the RIDA (Regulatory Inactivation of DnaA) and DDAH (*datA*-dependent DnaA^ATP^ hydrolysis) processes. During RIDA, the Hda protein complexed with the DNA loaded β-clamp stimulates the intrinsic ATPase activity of DnaA thereby converting DnaA^ATP^ to the non-active DnaA^ADP^ [10, 11]. DDAH is less efficient and takes place at the *datA* locus where a complex of *datA* and IHF promotes DnaA^ATP^ hydrolysis [12]. If extra initiation events are triggered by loss of RIDA or by conditional mutations in DnaA [13], DNA strand breaks progressively accumulate, eventually resulting in cell death. It was shown that the lethal accumulation of strand breaks in such cells resulted from replication forks encountering DNA damage repair intermediates, particularly resulting from oxidative damage to the DNA during normal aerobic growth. Therefore, growth could be restored in the absence of oxygen or by removing the predominant glycosylase of oxidized bases [14].

**Fig 1:**
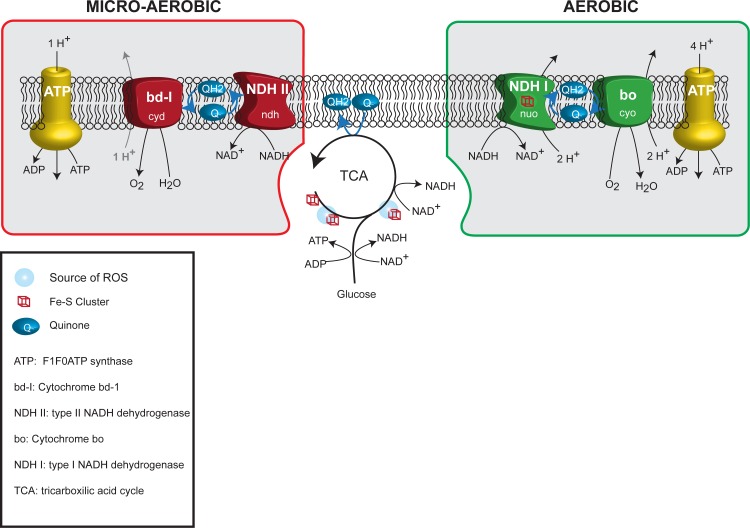
Simplistic representation of oxidative phosphorylation pathways in *E*. *coli*. The aerobic (green frame) and micro-aerobic (red frame) respiratory chain are represented together with the tricarboxylic acid cycle and glycolysis. Iron sulfur clusters and known sources of ROS associated with these pathways are also shown.

During aerobic growth, a proton gradient is generated by a respiratory chain made of the type I dehydrogenases containing iron-sulfur proteins and the cytochrome bo that is efficient and has low affinity for oxygen (Fig 1)[15]. It is controversial how Reactive Oxygen Species (ROS) are produced in *E*. *coli* [16]. Respiration *per se* is not generating ROS [17, 18]. In contrast, respiration is thought to limit ROS formation by pulling away electrons from potential ROS-sources [19]. For example, mutants lacking NAD dehydrogenases I and II or cytochrome oxidases bo and bd-1 produces more H_2_O_2_. The main cellular sources of ROS are thought to be free iron, flavins and iron sulfur cluster proteins with the dehydratase enzymes of the TCA cycle as the main culprits [20].

During micro-aerobic growth, another set of proteins which are less efficient in generating a proton gradient dominates the respiratory chain. These consist of a copper containing dehydrogenase (NDHII) and the cytochrome bd-I terminal oxidase that has a high affinity for oxygen (Fig 1). This micro-aerobic chain is also predominant when iron is scarce or during oxidative stress [21] (for review [15, 22]). During these stress conditions the cytochrome bd-I is thought to act as an electron sink to reduce the ROS level [19].

We previously identified two mutations in the *iscU* and *fre* genes (*iscUC63F* and *fre*Δ*68)* that suppress RIDA deficiency [23]. These genes encode an Iron-Sulphur cluster scaffold protein and Flavin reductase, respectively. Here, we provide evidence that the mechanism of suppression is not linked to DnaA or replication initiation activity. Global transcription analysis of *iscUC63F* and *fre*Δ*68* cells showed that genes encoding enzymes of the TCA cycle were down regulated in both mutants while respiration was altered to favor the use of the micro-aerobic respiratory chain. Therefore, these two mutants may tolerate overinitiation in a manner similar to cells growing in the absence of oxygen. For the *fre*Δ*68* mutant, we show that the ArcA regulon plays a crucial role for suppression in part by upregulating *cyd* transcription to overproduce cytochrome bd-1 [24, 25].

## Results

### The *iscUC63F* and *freΔ68* mutations suppress Hda deficiency without reducing initiations from *oriC*

*Hda* mutant cells accumulate strand breaks under aerobic conditions resulting in progressive growth inhibition, and loss of colony forming ability, unless a suppressor mutation is acquired [23, 26, 27]. The nature of several suppressor mutations was previously identified [23]. One suppressor is a missense mutation in *iscU* resulting in cysteine being replaced with phenylalanine at position 63 of the scaffold protein for assembly of iron sulfur clusters, IscU (IscUC63F). Iron sulfur clusters are used in a variety of cellular activities such as respiration, amino acid synthesis and DNA repair. A second suppressor is a 380 bp deletion between two imperfect repeats starting at position 497bp after the start codon of the *fre* gene and ending in the intergenic *fre*-*fadA* region. This results in a premature stop codon and loss of the 68 C-terminal amino acids of the flavin reductase (FreΔ68). The Flavin reductase catalyzes the reduction of free flavins by NAD(P)H. It is thought that Fre accounts for more than 80 percent of the free Flavin reduction *in E*. *coli* [28, 29] and may serve as a general cytosolic source of electrons [30].

**Fig 2:**
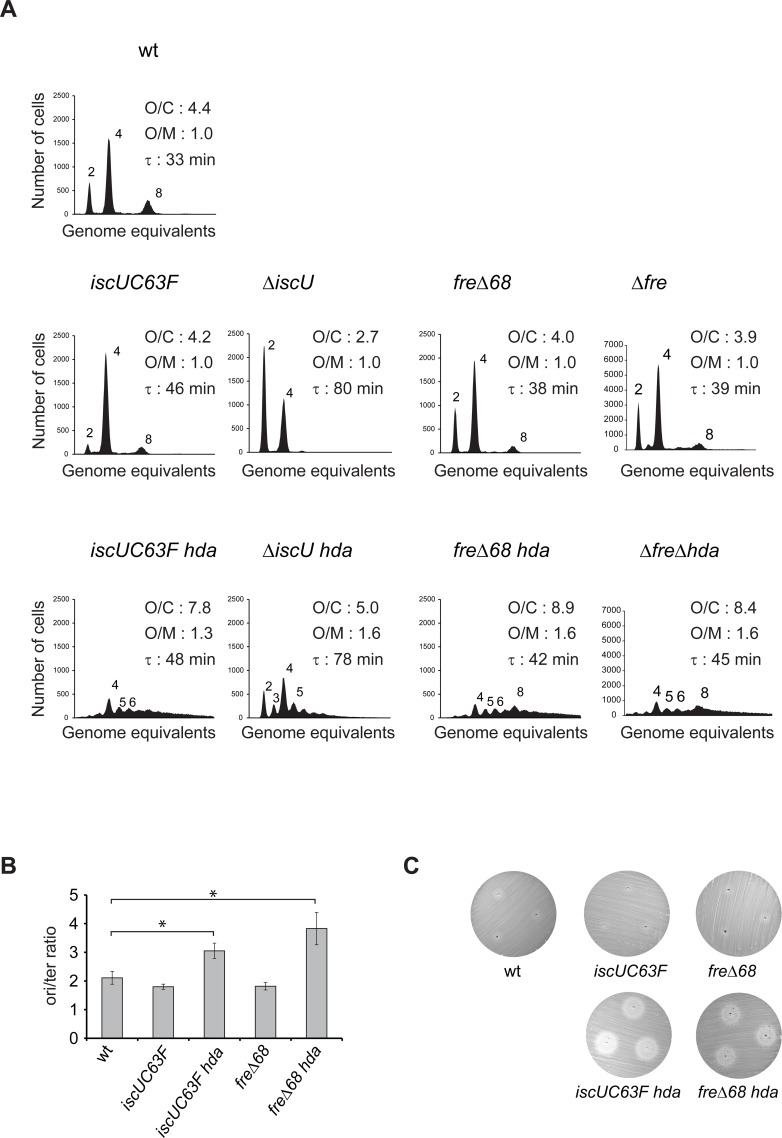
Cell cycle profile of *iscU* and *fre* mutants. A) Cells were grown exponentially in AB medium supplemented with 0.2% glucose and 0.5% casamino acids and treated with rifampicin and cephalexin prior to flow cytometric analysis. Each panel represents a minimum of 30000 cells. The average *ori*/cell (O/C), *ori*/mass (O/M) relative to wild-type and mass doubling time (τ) are inserted in the histograms. B) *ori*/*ter* ratio determined by qPCR analysis. Shown is the mean ± s.d. (n = 3),* = p<0.05. C) *IscUC63F* and *fre*Δ*68* mutations do not alter the sensitivity to hydroxyurea. Sensitivity to hydroxyurea is tested by a diffusion assay on cells plated on LB agar medium. Cells are plated evenly on LB plates, 3μl of 500mM HU was introduced into 3 separated holes and plates incubated at 37°C for 16h.

Cells carrying *iscUC63F* and *fre*Δ68 mutations grew somewhat slower than the wild-type (Fig 2A). Analysis of chromosome replication by flow cytometry revealed that both mutants initiated in synchrony with a small reduction in numbers of origins per cell corresponding to the slower growth rate but the origin concentration remained unchanged (Fig 2A). Loss of RIDA activity by deletion of *hda* (referred to as loss of RIDA throughout this work), resulted in initiation asynchrony, increased the average number of origins per cell from around 4 to 7.8 and 8.9 for *iscUC63F* and *fre*Δ68, respectively, and increased the origin concentration (Fig 2A). Note that these are minimum estimates for numbers of origins per cell as runout was too poor to allow for an exact enumeration. This shows that the *iscUC63F* and *fre*Δ68 mutations restore growth of Hda deficient cells despite of continued overinitiation.

In order to determine whether the *iscUC63F* and *fre*Δ68 mutations resulted in change or loss of function of their respective proteins we proceeded to delete *iscU* and *fre* from otherwise wild-type cells. Complete loss of Flavin reductase resulted in a cell cycle profile similar to that observed for the *fre*Δ68 mutant cells suggesting that the FreΔ68 protein is not functional (Fig 2A). Loss of *iscU* on the other hand resulted in cells that grew slower than *iscUC63F* cells and that had a reduced number of cellular origins (Fig 2A). Loss of *iscU* restored growth of Hda deficient cells while these continued to overinitiate in an asynchronous manner (Fig 2A). The relative increase in origin concentration caused by the deletion of *hda* in Δ*iscU* relative to *iscUC63F* may be a simple consequence of a better runout in Δ*iscU* cells. This suggests that Δ*iscU* is a better suppressor than *iscUC63F* but altogether the data indicate that the IscUC63F protein is partly functional which is in agreement with an earlier report showing that an IscUC63A mutant protein is able to form and transfer [2Fe-2S] clusters with reduced activity compared to the wild-type enzyme [31]. The *ori*/*ter* ratio of *iscUC63F* and *fre*Δ68 also increased from a level of 1.8 similar to wild-type cells, to about 3 and 3.8 upon loss of Hda (Fig 2B). Taken together, the origin concentration and *ori*/*ter* ratios indicates that replication fork processivity was uncompromised in Hda deficient cells also carrying *iscUC63F* and *fre*Δ68 mutations, despite of overinitiation. A Pulse Field Gel Electrophoresis analysis confirmed this, as the levels of fragmented chromosomes in *hda iscUC63F* and *hda fre*Δ68 was greatly reduced compared to Hda deficient cells (S1 Fig).

### *iscUC63F* and *fre*Δ*68* mutations suppress the loss of Hda in a growth rate independent manner

We previously suggested that formation of lethal double strand breaks in an *hda* mutant is intimately linked to the number of replicative forks moving on the chromosome [14]. The number of ongoing replication forks in a cell can be reduced simply by reducing growth rate. We therefore streaked anaerobically generated *hda* cells on minimal medium plates supplemented with glycerol (S2A Fig) and with glucose and casamino acids (S2B Fig) and incubated aerobically at 37°C. *Hda* cells failed to form colonies on minimal plates with glucose and casamino acids, but formed colonies as well as wild-type cells on minimal medium plates supplemented with glycerol. To ensure that the cells had not accumulated suppressor mutations, glycerol grown *hda* cells were restreaked on LB agar in aerobic condition (S2C Fig). These cells failed to form colonies, implying that the glycerol selected *hda* clones had not acquired suppressor mutations. The growth rate of *hda* cells in minimal medium supplemented with glycerol was similar to that of the wild-type but initiation of replication was asynchronous and origin concentration was increased (S2D Fig). *Hda* cells were then shifted from growth in glycerol to glucose and casamino acids (S2E Fig) or LB medium (S2F Fig) for three mass doubling time. As expected, this resulted in an increased origin concentration (relative origin per mass equal to 1.7 or 1.8) reminiscent of the shift observed between anaerobic to aerobic conditions [14]. Overall, these observations indicate that *hda* is dispensable in slow growing cells, consistent with what has been observed for other overinitiation mutants [32].

Cells carrying *iscUC63F* and *fre*Δ68 mutations had a reduced growth rate in minimal medium supplemented with glucose and casamino acids relative to wild-type cells (46 and 38 versus 33 minutes, respectively; Fig 2A) and contained fewer origins and hence fewer ongoing replication forks per cell. A possibility was therefore that the ability of these mutants to suppress Hda deficiency resulted from slow growth. The *iscUC63F* and *fre*Δ68 mutations were originally isolated as *hda* suppressors from cultures grown in LB [23, 26]. In this medium, the mutants grew with doubling times of 30 and 21 minutes, respectively, and contained a high number of origins and ongoing replication forks per cell, yet loss of Hda was tolerated (S3 Fig). A reduced growth rate is therefore not the main mechanism of suppression in these cells.

### Hda deficient *iscUC63F* and *fre*Δ*68* cells are sensitive to hydroxyurea

Since overexpression of Ribonucleotide Reductase Ia (RNRIa) encoded by *nrdAB* genes or RNRIb encoded by *nrdEF* genes were previously shown to suppress the loss of *hda* [27, 33], we tested whether an increase in amount or activity of these enzymes is present in *iscUC63F* and *fre*Δ*68* cells. To assess the Ribonucleotide Reductase activity we made use of the RNR inhibitor hydroxyurea (HU). The sensitivities to hydroxyurea of *iscUC63F* and *fre*Δ68 mutant cells were similar to wild-type in our assay (Fig 2C) although a *fre* mutant has been previously shown to have a 10 to 50% growth reduction in the presence of 10 to 40 mM HU. In the absence of Hda, both the *iscUC63F* and *fre*Δ68 mutants became hypersensitive to HU, indicating that the RNR activity is indeed critical for survival and that the dNTP pool is limiting in such cells.

### Global transcription profile of *iscUC63F* and *fre*Δ*68* cells

**Fig 3:**
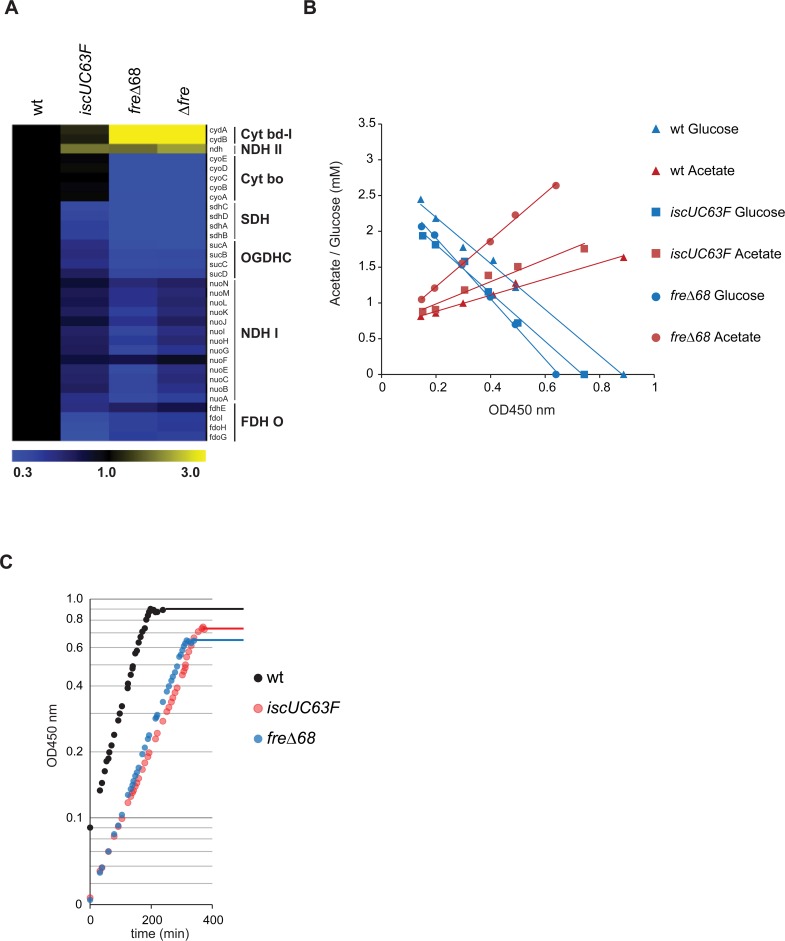
The respiro-fermentative metabolism of *IscUC63F* and *fre*Δ68 mutant cells. A) Expression profiles of genes involved in the respiratory chain as obtained by whole genome microarray. Expression intensities relative to wild-type are displayed by a two color gradient from blue (low) to yellow (high). Shown, the expression profile of operons encoding succinate dehydrogenase (SDH), 2-oxoglutarate dehydrogenase (OGDHC), NADH dehydrogenase I (NDH-I) and II (NDH-II), formate dehydrogenase (FDH O), cytochrome o oxidase (Cyt bo) and bd-1 (Cyt bd-1). B) Measurement of glucose consumption and acetate production during growth in glucose limited medium (AB minimal medium supplemented with 0.04% glucose). The plot shows the concentration of glucose (blue symbols), and acetate (red symbols) over optical density (no lactate, formate, citrate, ethanol and succinate were detected). Samples for final optical density for wild-type, *IscUC63F* and *fre*Δ68 were measured at growth arrest (OD_450_ nm 0.88, 0.75, 0.64 respectively). C) Growth of wild-type, *IscUC63F* and *fre*Δ*68* cultures in AB minimal medium supplemented with 0.04% glucose.

To gain insight into the mechanism of *hda* suppression by the *iscUC63F* and *fre*Δ68 mutations we performed a global transcription analysis of cells grown in minimal medium supplemented with glucose and casamino acids using microarrays. The two mutants had both changes in gene expression that were specific to each mutant and changes that were in common. Genes that were specifically upregulated in the *iscUC63F* mutant included those encoded by the *iscRSUA hsbB* and *sufABCDSE* operons (S1 Table) which is in agreement with a poorly functioning IscUC63F protein [34–36]. Note that we also observe an increased expression of *nrdHEIF* (see discussion). In the *fre*Δ68 strain, specifically the *cyd* operon encoding cytochrome bd-1 was upregulated and the majority of dehydrogenases were downregulated (S2 Table). Overproduction of Fre has little consequences on the transcription profile (S2 Table). Similarities between *iscUC63F*, *fre*Δ*68* and Δ*fre* cells included expression of genes whose products are involved in cellular respiration (Fig 3A, S1 and S2 Tables). The *sdh* operon encoding succinate dehydrogenase (succinate-coenzyme Q reductase; SDH), the *suc* operon encoding 2-oxoglutarate dehydrogenase (OGDHC), the *nuo* operon encoding NADH dehydrogenase I (NDH-I)and the *fdo* operon encoding formate dehydrogenase (FDH O), most of which contain [Fe-S] clusters, are all part of the respiratory chain and are down-regulated. In addition the *cyo* genes encoding cytochrome o oxidase (Cyt bo) (S2 Table), are downregulated in *fre*Δ68 and *fre* deleted cells (Fig 3A, S1 and S2 Tables). On the other hand *ndh* encoding the type II NADH dehydrogenase (NDH-II), which is a metalo enzyme dependent on copper, is overexpressed in *iscUC63F*, *fre*Δ*68* and Δ*fre* cells. This is in accordance with a report showing that in *iscU* mutants, NDH-I activity is reduced to background level while NDH-II activity is increased [37]. Finally, the *cydAB* genes encoding cytochrome bd-1 (Cyt bdI) (S2 Table) and to some extent the genes encoding *appBC* cytochrome bd-2 (Cyt bdII) are overproduced in *fre*Δ68 and *fre* deleted cells. The microarray results are coming from single experiments, therefore, the expression patterns of *cydA*, *sdhD* and *cyoA* genes were confirmed by RT-qPCR (S3 Table).

Overall these expression data points towards *IscUC63F* and *fre*Δ*68* cells being shifted from a normal aerobic respiration pattern towards what would be characteristic of cells growing under micro-aerobic or iron limited conditions (Fig 1).

### A metabolic shift in *IscUC63F* and *fre*Δ*68* cells

We proceeded to determine whether *IscUC63F* and *fre*Δ*68* cells had their metabolism shifted from the normal aerobic respiration towards the less efficient Cytochrome bd-1 dependent respiratory pathway or even fermentation, despite of growing in aerobic conditions. In order to obtain precise measurements of metabolites, cells were grown in minimal medium supplemented with 0.04% glucose. Surprisingly, we found that the ATP/ADP ratio was increased by 50–60% in both *IscUC63F* and *fre*Δ*68* cells (Table 1). This rules out that these mutations suppress overinitiation by lowering the ATP/ADP ratio which in turn could lower the DnaA^ATP^/DnaA^ADP^ ratio, as both nucleotides bind DnaA with similar affinities [3]. The NADH/NAD^+^ ratio was similar in wild-type and *IscUC63F* cells but was increased by more than two-fold in *fre*Δ*68* cells. The production of acetate per mole of glucose was also increased by 100% and 40% in *fre*Δ*68* and *IscUC63F* cells, respectively (Table 1; Fig 3B). Formate, ethanol, succinate, citrate and lactate were not produced in any of the strains. The *IscUC63F* and *fre*Δ*68* strains also reached lower optical density when glucose consumption ended, i.e. had a lower yield of biomass per mole of glucose (Fig 3C).

These results agree with the microarray data (Fig 3A) and show that the metabolism of *iscUC63F* and *fre*Δ*68* cells is partly re-routed toward acetate production as would be expected when the TCA cycle is downregulated. The increase in acetate production in *fre*Δ*68* cells is consistent with a high NADH/NAD^+^ ratio [38, 39], which in turn agrees with the suggestion that flavin reductase accounts for a significant part of the NADH cellular oxidation [28]. The high ATP/ADP ratio in *iscUC63F* and *fre*Δ*68* suggests that anabolic reactions are reduced in the strains. This increased ratio could also decrease the flux in the TCA cycle by allosteric inhibition [40]. Our microarray analysis indicates that respiration in *iscUC63F* and *fre*Δ*68* cells could be shifted towards the less efficient Cytochrome bd-1, therefore it is expected that ATP production through oxidative phosphorylation is reduced in these strains. The oxygen consumption of *iscUC63F* and *fre*Δ*68* cells was decreased somewhat as expected (Table 1).

**Table 1:** Metabolic parameters of *IscUC63F* and *fre*Δ*68* mutant cells.

	Wild-type	*IscUC63F*	*fre*Δ*68*
Doubling time (min)^1^	60	89	81
Growth rate (ln2/t_d_)/h^1^	0.70	0.47	0.51
Specific rate of Glucose consumption ^1^ (mmol/gDW/h)	11.80	8.28	11.44
Specific rate of Acetate production ^1^ (mmol/gDW/h)	4.15	3.80	8.82
Acetate yield on Glucose (mol/mol) ^1^	0.35	0.46	0.77
Biomass yield on Glucose (gDW/mmol) ^1^	0.059	0.056	0.045
Relative Respiration rate (%) ^2^ † (% oxygen/gDW/min)	100 (7.6)	77.2 (5.5)	63.8 (4.0)
ATP/ADP ratio^1^	7.5 (0.7)	12.1 (2.0)	11.4 (1.9)
NADH/NAD^+^ ratio^1^	0.2*	0.2*	0.5*

*Average of two samples: wild-type = (0.24 + 0.18), *IscUC63F* = (0.21 + 0.21), *fre*Δ*68* = (0.55 + 0.48)

^1^Cells were grown in AB medium supplemented with 0.04% glucose at 37°C.

^2^Cells were grown in AB medium supplemented with 0.2% glucose at 37°C.

† wild-type respiration rate = 885% oxygen/gDW/min.

### Role of the Arc regulon in *hda* suppression

The transcription profiles of *fre*Δ*68* and Δ*fre* cells are by and large consistent with the activation of the ArcA regulon [25, 41]. The ArcBA (anoxic redox control) two-component system, senses the redox state of the cell and reprograms the metabolism to increase the availability of NAD^+^ required for glycolysis. ArcA activity is coupled to the NADH/NAD^+^ balance and conditions that artificially increase the ratio NADH/NAD^+^ have been shown to activate the ArcA regulon [38].

When oxygen becomes limiting, ArcA represses genes involved in the generation of NADH, including those specifying enzymes of the TCA cycle, while genes encoding enzymes involved in fermentative regeneration of NADH into NAD^+^ are activated [25].

**Fig 4:**
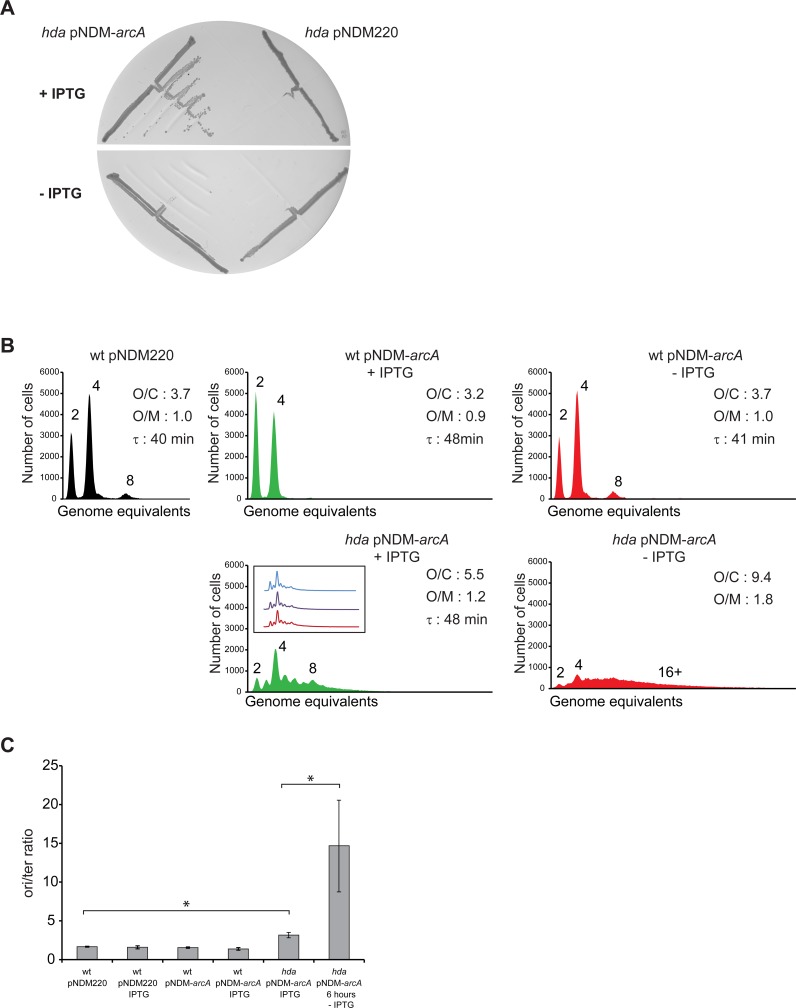
Hda deficiency is suppressed by ArcA overexpression. A) The *hda*::*cat* mutation was introduced into wild-type cells containing plasmid pNDM220 or pNDM-*arcA* under anaerobic conditions. Cells were restreaked on LB plates with or without the IPTG and incubated aerobically at 32°C for 24 hours. B) Cells were grown exponentially at 32°C in AB medium supplemented with 0.2% glucose and 0.5% casamino acids and treated with rifampicin and cephalexin prior to flow cytometric analysis. When indicated cells were grown with 1mM IPTG. The flow diagram of “*hda*/pNDM-*arcA* -IPTG” was obtained from cells grown in presence of 1mM IPTG then washed and regrown in absence of IPTG for 6 hours. Each panel represents a minimum of 30000 cells. The average *ori*/cell (O/C), *ori*/mass (O/M) relative to wt/pNDM220 and mass doubling time (τ) are inserted in the histograms. C) The cellular *ori*/*ter* ratio determined by qPCR analysis. Shown is the mean ± s.d (n = 3),* = p<0.05.

We cloned *arcA* under control of the IPTG regulated promoter pA1/O4/O3 promoter [42] in the R1 based plasmid pNDM220 [43] and proceeded to delete *hda* in the presence of IPTG. Restreaking of the resultant colonies indicated that colony formation depended on IPTG (Fig 4A) and suggests that ArcA is necessary for *fre*Δ*68* dependent suppression of Hda deficiency. Flow cytometry analysis revealed that initiation of replication in independent clones of *hda* cells overexpressing *arcA* was more frequent and asynchronous than in wild-type cells (Fig 4B). Similar to what was observed for *fre*Δ*68*Δ*hda* cells (Fig 2), the *ori*/*ter* ratio was somewhat increased (Fig 4C). When ArcA was depleted by shifting the cells to a medium lacking IPTG for six hours, the number of origins per cell increased dramatically as did the *ori*/*ter* ratio (Fig 4B and 4C). The relative origin concentration was also increased. This indicates that cells were not able to complete chromosome replication, probably due to DNA damage associated arrest or collapse of replication forks, while initiation of replication remained unperturbed [14].

Altogether these results indicate that the *fre*Δ*68* mutation results in a high NADH/NAD^+^ ratio which activates the ArcA regulon to shift cells from the normal aerobic respiration towards the less efficient Cytochrome bd-1 dependent respiratory pathway, despite of growing in aerobic conditions. In the absence of ArcA overproduction, initiation of replication continued but cells were not able to complete chromosome replication.

### Loss of ATPase activity suppress RIDA (Hda) Deficiency

**Fig 5:**
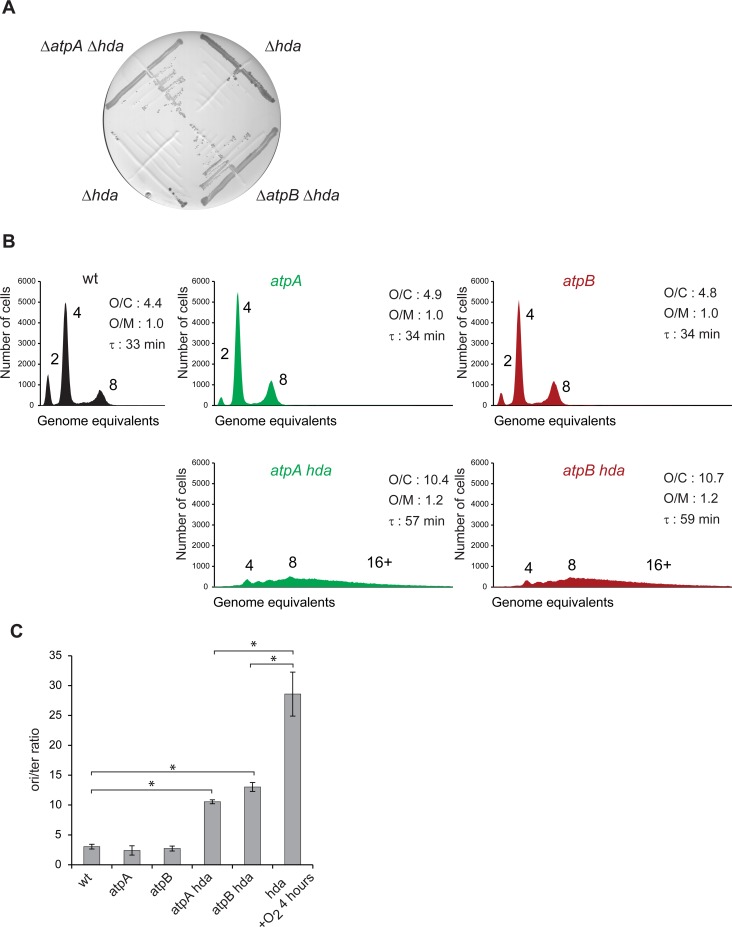
Loss of ATP synthase restores growth of Hda deficient cells. A) The *hda*::*cat* mutation was introduced into wild-type,Δ*atpA* and Δ*atpB* cells under anaerobic conditions, restreaked on LB plates and incubated under aerobic and anaerobic conditions. B) Wild-type, *atpA*, *atpB*, *atpA hda* and *atpB hda* mutants cells were grown exponentially in AB medium supplemented with 0.2% glucose and 0.5% casamino acids and treated with rifampicin and cephalexin prior to flow cytometric analysis. Each panel represents a minimum of 30000 cells. The average *ori*/cell (O/C), *ori*/mass (O/M) relative to wild-type and mass doubling time (τ) are inserted in the histograms. C) The cellular *ori*/*ter* ratio were determined by qPCR analysis. Shown is the mean ± s.d (n = 3),* = p<0.05.

We proceeded to mimic the metabolism of fermenting cells while growing aerobically by deleting the *atpA* or *atpB* genes encoding the F_1_F_0_ ATP synthase. In such cells the respiration rate increases but is uncoupled, i.e. does not result in ATP production end energy is solely produced by fermentation. Consequently the cellular ATP/ADP ratio is reduced to about one third of the wild-type level [44]. In ATPase deficient cells, cytochrome bo remains unchanged, cytochrome bd-1 and NDHII are overproduced while the TCA cycle is repressed leading to a redirection of the glycolytic flux towards acetate production [45]. The composition of the respiratory chain used by ATPase deficient cells thus resembles the one used under micro-aerobic conditions and the one used by *fre*Δ*68* cells (Fig 1), which is thought to favor NAD^+^ regenerations [45]. We tested whether cells lacking ATPase could tolerate the loss of Hda similar to *fre*Δ*68* cells. *Hda* was deleted in Δ*atpA* or Δ*atpB* cells under anaerobic conditions. Restreaking in the presence of oxygen demonstrated that Hda is indeed dispensable for growth of ATPase deficient cells (Fig 5A). Cells deleted for *atpA* or *atpB* had doubling times and cell cycle parameters similar to wild-type cells (Fig 5B). A further loss of *hda* in the *atpA* and *atpB* mutant cells resulted in an elevated number of chromosomes per cell, an increased origin concentration and an increased *ori*/*ter* ratio (Fig 5C) relative to wild-type. Note that although increased, the *ori*/*ter* ratio in *hda atpA* and *hda atpB* is still lower than that of *hda* cells shifted from anaerobic to aerobic growth for 4 hours (Fig 5C), indicating that replication fork progression is affected in *hda atpA* and *hda atpB* but to a tolerable level. Again this demonstrates that the cellular ATP/ADP ratio has little influence on replication initiation, and that loss of ATPase function permits cells to survive despite of overinitiation similar to what we observed for *iscUC63F* and *fre*Δ*68* cells.

### Cytochrome bd-1 function is critical for survival in absence of *hdA*

The cytochrome bd-1 complex is upregulated in *fre* (S2 and S3 Tables) and ATPase mutants [45] and may provide protection against ROS because of its high affinity to O_2_ and potential peroxidase activity [21, 46–48].

**Fig 6:**
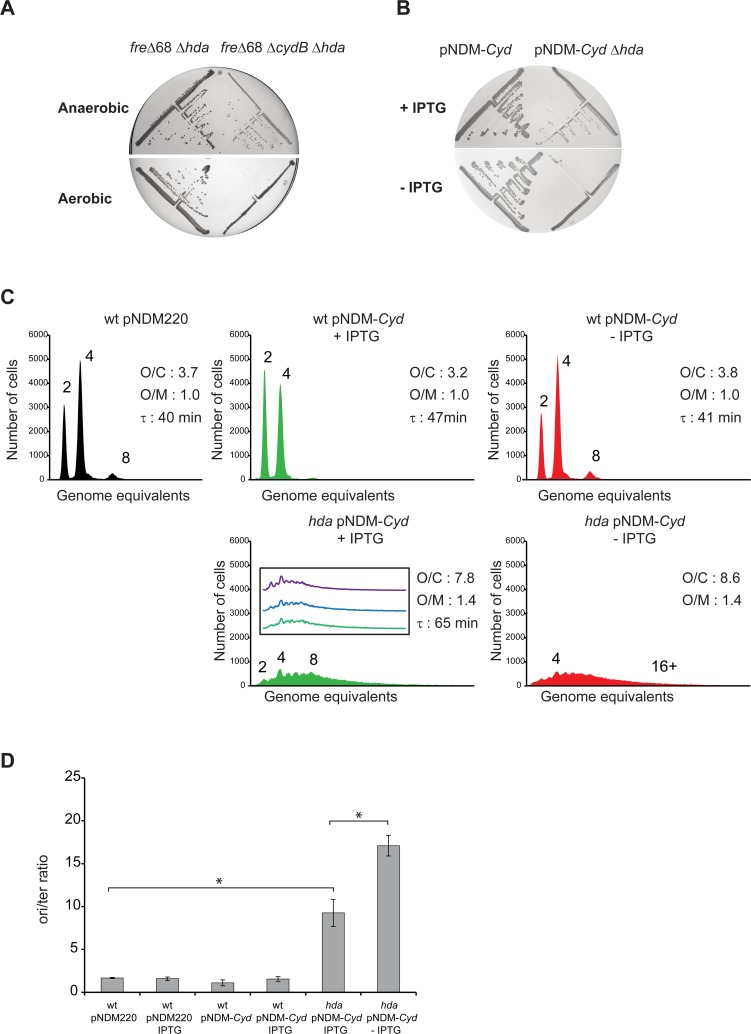
Cytochrome bd-1 function is required for *fre*Δ68 survival in absence of Hda. A) The *hda*::*cat* mutation was introduced into *fre*Δ*68* and *fre*Δ*68*Δ*cydB* cells under anaerobic conditions, restreaked on LB plates under aerobic and anaerobic conditions. Plates were incubated at 32°C for 24 hours. B) The *hda*::*cat* mutation was introduced into wild-type cells containing plasmid pNDM220 or pNDM220-*cyd* under anaerobic conditions. Cells were restreaked on LB plates with or without the IPTG and incubated aerobically at 32°C for 24 hours. C) Cells were grown exponentially at 32°C in AB medium supplemented with 0.2% glucose and 0.5% casamino acids and treated with rifampicin and cephalexin prior to flow cytometric analysis. When indicated cells were grown with 1mM IPTG. The flow diagram of “hda/pNDM-*cyd* -IPTG” was obtained from cells grown in presence of 1mM IPTG that was washed and regrown in absence of IPTG for 16 hours. The average *ori*/cell (O/C), *ori*/mass (O/M) relative to wild-type pNDM220 and mass doubling time (τ) are inserted in the histograms. D) The cellular *ori*/*ter* ratios were determined by qPCR analysis. Shown is the mean ± s.d (n = 3),* = p<0.05.

We deleted the *cydB* gene, essential for the cytochrome bd-1 activity, in wild-type, *fre*Δ*68* and *fre*Δ*68 hda*, mutant at 32°C under anaerobic conditions. The low temperature was chosen as *cydB* mutants grow poorly at 37°C [49]. Colonies were restreaked anaerobically and aerobically and revealed that loss of *cydB* in the *fre*Δ*68 hda* mutant led to severe growth inhibition under aerobic conditions (Fig 6A). Therefore cytochrome bd-1 is instrumental in the *fre*Δ*68* mechanism of *hda* suppression.

We also deleted *cydB* in *atpA hda* and *atpB hda* mutants and similar to *fre*Δ*68 hda*, the cells were unable to grow aerobically showing that cytochrome bd-1 is also essential for *hda* suppression through loss of ATPase activity (S4 Fig). Although not overproduced in *iscU* mutants the cytochrome bd-1 was also found essential when *hda iscUC63F* cells were grown aerobically, indicating its function is critical despite the presence of the cytochrome bo (S4 Fig).

We proceeded to overproduce cytochrome bd-1 (pNDM-Cyd) and found that *hda* could be deleted in such cells without loss of ability to form colonies (Fig 6B) although the small colony size suggested that pNDM-Cyd was a relatively poor *hda* suppressor. In agreement with this, independent clones of *hda* cells overexpressing cytochrome bd-1 grow with a relative high cellular origin concentration but were all similar when analyzed by flow cytometry (Fig 6C). When depleted for cytochrome bd-1, the origin concentration increased overtime although this was difficult to assess due to incomplete runout after rifampicin and cephalexin treatment. The *ori*/*ter* ratio was increased from about 10 to about 20 following 16 hours of cytochrome bd-1 depletion (Fig 6D).

### ROS levels in *iscUC63F* and *fre*Δ*68* cells

In *E*. *coli*, a series of dedicated enzymes detoxify endogenously generated reactive oxygen species. Among the different systems that sense ROS, the OxyR system that perceives and reacts to the threat of hydrogen peroxide accumulation is the best understood. To preserve the cellular homeostasis in case of an H_2_O_2_ assault, OxyR activates the transcription of genes involved in protective or detoxifying processes [50]. Although OxyR activation is mostly considered a sensor of extracellular elevated H_2_O_2_ levels [50], down regulation of the OxyR regulon in *iscUC63F*, *fre*Δ68 cells could imply that these cells generate less H_2_O_2_ endogenously. The microarray data did however not reveal down regulation of the OxyR regulon in *iscUC63F*, *fre*Δ68 cells (S5 Fig). As microarray data may not reveal minor differences in gene expression we decided determine expression of *katG*, encoding a catalase strongly induced by OxyR, by using a *katG*::*lacZ* transcriptional fusion carried on the chromosome [51]. We did not observe major changes in *katG* expression in *iscUC63F* and *fre*Δ*68* (S6 Fig) which could indicate that the cytoplasmic level of H_2_O_2_ is not reduced in the mutants. We did not find the Sox regulon is affected in *iscUC63F* and *fre*Δ*68* cells either (S5 Fig; S1 Appendix). Although this may indicate that superoxide levels are not changed in the mutants, the SoxS/R system is now believed to react to redox cycling molecules rather than superoxide directly [52].

We also assessed the impact of superoxide dismutase *sodA* and *sodB* mutations on survival of overinitiating cells. Deletion of *sodA* had little effect on the growth of wild-type, *hda fre*Δ*68* cells while deletion of *sodB* appeared deleterious (S7 Fig). This implies that superoxide accumulation is toxic in overinitiating cells but tolerated in wild-type cells. SodA and SodB differ especially by the nature of the metal cofactor Mn and Fe respectively [53]. MnSodA is involved in oxidative stress response, while FeSodB normally provides the generic scavenging activity. The sensitivity of *hda fre*Δ*68* to the loss of *sodB* only may reflect the fact that *sodA* is repressed by ArcA directly at the transcriptional level and potentially also indirectly at the translational level through the small RNA FnrS (not present in our microarray) [54]. Altogether, this indicates that *sodB hda fre*Δ*68* may be low in cytoplasmic superoxide dismutase activity, a stressing situation already for wild-type cells.

## Discussion

In *E*. *coli* excessive initiations from *oriC* result in progressive growth inhibition due to the accumulation of DNA strand breaks [13, 14]. The isolation and characterization of second site suppressor mutations that overcome this inviability has revealed that they fall into two categories. The first category includes mutations that reduce *oriC* activity and are found in *oriC* itself [55], in *dnaA* or in genes that affect DnaA function or activity [23, 27, 33, 56–59]. The second category of suppressor mutations have in common that they do not reduce initiation frequency but allow cells to survive in spite of overinitiation. These mutations facilitate replication fork progression along the chromosome either by increasing the size of the dNTP pool (overexpression of Ribonucleotide Reductase; [27, 33]), by altering the DNA topology (ex: mutation in *hns*; [60]), or by limiting the repair of DNA damages (i.e. *mutM*;[14]). Here we characterize three *hda* suppressor mutations in *iscU*, *fre* and *atpAB* that seemingly promote viability by limiting oxidative damage to DNA.

### An altered energy charge does not affect replication initiation from *oriC*

**Fig 7:**
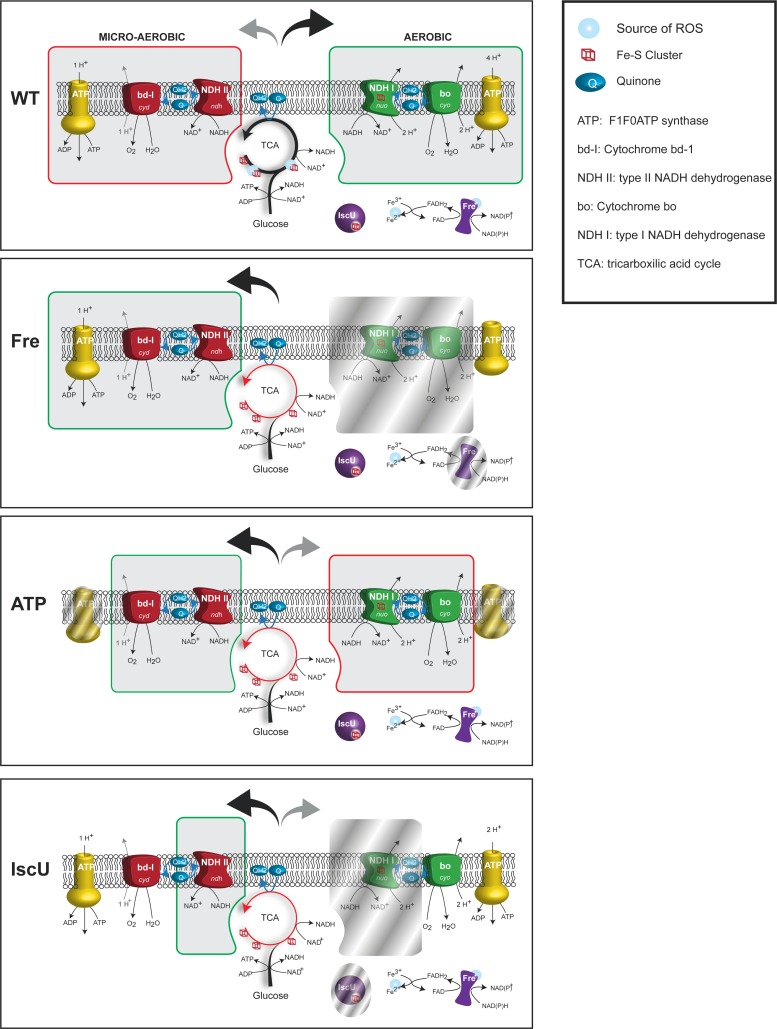
Respiratory pathways in cells carrying *hda* suppressor mutations. Representation of the TCA cycle and the respiratory chain components of cells grown aerobically in presence of glucose. For each strain, the predominant respiratory path is framed in green while secondary or repressed paths are framed in red or shaded respectively.

The *iscUC63F*, *fre*Δ*68* and *atpAB* mutants all have in common that the TCA cycle is downregulated and all, or parts of the micro-aerobic respiratory chain is upregulated (Fig 7). While the micro-aerobic respiratory chain is relatively inefficient in generating a proton gradient (Fig 7), the ATPase mutants cannot even utilize the proton gradient for ATP production and one might therefore suspect that the ATP/ADP ratio is lowered in all of these cell types. Because DnaA has the same affinity for ATP and ADP [3] a lowered ATP/ADP ratio would result in generation of less DnaA^ATP^ upon rejuvenation or *de novo* synthesis, which could contribute to a lowered overall DnaA^ATP^/DnaA^ADP^ ratio. In turn, this could explain the ability of the *iscUC63F* mutation to suppress RIDA deficiency by lowering initiations from *oriC*. However, two lines of evidence argue against this. First, we found that initiation of replication was not significantly affected in any of the mutants with respect to origin concentration and initiation synchrony. Second, the ATP/ADP ratio is actually increased in *iscUC63F* and *fre*Δ*68*, and only reduced in *atpAB* mutants [44].This argues that the changes in ATP/ADP ratio observed here has little influence on initiation frequency. This also implies that either the ATP/ADP ratio must be changed more dramatically to affect initiation of replication or that other mechanisms dedicated to maintain the balance DnaA^ATP^/DnaA^ADP^ such as RIDA, DDAH, and DARS mediated rejuvenation counteract any gross variations in the cellular ATP/ADP ratio to maintain an initiation frequency that is tightly coupled to cell mass increase.

It should also be noted that the energy charge of *E*. *coli* is normally relatively invariable [61–63] at different growth rates and whether cells are grown aerobically or anaerobically. This is thought to be controlled by changes in the glycolysis flux in response to the demands in ATP [44]. In wild-type cells, the cellular energy charge is only affected during adaptation to environmental changes or stress [62, 63].

### Increased dNTP synthesis is not the primary suppression mechanism in *iscUC63F* and *fre*Δ*68*

Overexpression of the Ribonucleotide Reductase is known to suppress the loss of *hda* [27, 33]. In *iscUC63F* cells we observed that transcription of *nrdHEIF* genes encoding RNR1b was increased about two-fold (S1 Table). RNRIa and RNRIb differ mostly in the dinuclear metal cluster required for their activity: a 2Fe-tyrosyl radical (RNRIa) or a 2Mn-tyrosyl radical (RNRIb). However, the activation of *nrdHEIF* transcription is assumed to palliate a deficiency of the RNRIa only during iron limitation or oxidative stress [64, 65] and requires a concomitant overexpression of the manganese transporter MntH [64] which is not upregulated in the *iscUC63F* mutant (S1 Table). The RNRIa activity is likely to be reduced in *iscUC63F* mutant cells because RNR1a function is normally helped by the [Fe-S] cluster protein YfaE. The net result of the *iscUC63F* mutation is therefore unlikely to be cells with an increased dNTP pool. This is corroborated by a similar sensitivity of *IscUC63F* cells to the RNR inhibitor hydroxyurea as wild-type cells. Importantly, Hda deficient cells suppressed by overproduction of RNRIa or RNRIb no longer overinitiate replication, i.e. the origin concentration is similar to wild-type. RNR overproduction therefore reduces *oriC* activity in *hda* cells [27]. This is quite different from the continued overinitiation of *hda iscUC63F* and *hda fre*Δ*68* cells, which suggest that suppression is not mediated through an increased dNTP level in these mutants. The efficient HU-mediated killing of Hda deficient *iscUC63F* cells also shows that RNR activity is critical for survival of these cells and therefore that the dNTP pool is still limiting. The situation is similar in the *fre*Δ*68* mutant. Fre is a well-known *in vitro* activator of RNRIa [66]. The *fre*Δ*68* mutant sensitivity to HU was similar to wild-type in our assay but *hda fre*Δ*68* cells were effectively killed by HU treatment. This implies that in absence of Hda, the dNTP pool is limiting in *iscUC63F* and *fre*Δ*68*, although we cannot exclude that the dNTP pool is larger in comparison to what would be found in Fre^+^ *hda* and IscU^+^ *hda* cells had they been viable.

### Survival through a decrease in cellular ROS production?

The enzymes of the TCA cycle are downregulated in *iscUC63F*, *fre*Δ*68* and *atpAB* mutants (Fig 7) resulting in reduced ROS production arising from the dehydratases [16]. ROS production is expected to be further reduced in *iscUC63F* cells as this mutant is partly defective in synthesis of the [Fe-S] clusters required for function of many of the dehydratases (Fig 7). ROS production is also likely to be further reduced in the *fre*Δ*68* mutant because the generation of FADH_2_ is affected in the flavin reductase mutant [28]. FADH_2_ reduces Fe^+++^ to Fe^++^ which participates in the Fenton reaction to generate ROS [17] (Fig 7).

The aerobic respiratory chain is also altered in the mutants (Fig 7). For the *iscUC63F* mutant, this is due to the lack of Fe-S cluster required for several key enzymes in the pathway along with a transcriptional reprograming towards expression of enzymes devoid of Fe-S (S1 Table). For *fre*Δ*68* and *atpAB* mutants, an increased NADH/NAD ratio triggers a transcriptional repression of major aerobic chain components while the micro-aerobic chain is induced, notably by overproduction of cytochrome bd-1 (S2 Table; [38]), an enzyme known to scavenge or even process ROS species (Fig 7) [48, 67–69]. Cytochrome bd-1 was, although not upregulated, also necessary for suppression of overinitiation in *IscUC63F* cells, indicating that cytochrome bd-1 plays an essential role for growth even when the aerobic terminal oxidase cytochrome bo is also expressed. The importance of cytochrome bd-1 was demonstrated by showing that overproduction of cytochrome bd-1 directly or indirectly through ArcA overproduction could suppress RIDA deficiency and by showing that cytochrome bd-1 was absolutely required for *fre*Δ*68* mediated suppression of RIDA deficiency. There are in principle two known ways that cytochrome bd-1 can act as ROS scavenger. First, through its peroxidase activity, where the cellular localization of cytochrome bd-1 is found would suggest that it sanitizes the periplasm or exogenously created H_2_O_2_. Second, and probably most relevant in this context, by oxidizing quinone’s, that are also a source of ROS [70], cytochrome bd-1 could maintain a flux of electrons, thereby preventing flavoproteins (and quinones) to retain electrons and adventitiously passed them on to O_2_. Because ArcA overproduction did not significantly reduce the origin concentration of wild-type cells it is unlikely that ArcA binding to *oriC* [71] contribute to its ability to suppress overinitiation.

We previously proposed that aerobic inviability of Hda deficient cells results from an increased number of replication forks of which some may encounter an intermediary in the repair of primarily 8-oxodG to create double stranded breaks [14]. As 8-oxodG arises by oxidation of guanine residues in the DNA, primarily by hydroxyl radicals [72], growth can be restored in anaerobic conditions or by removal the GO-repair system, that is responsible for repairing 8-oxodG lesions.

During aerobic growth hydrogen peroxide (or superoxide) is generated as a consequence of flavin auto oxidation in dehydratases such as aconitase and fumarase of the TCA cycle, a process that could be reduced in *fre*Δ*68* and *iscUC63F* cells, due to down regulation of the flavoproteins in *fre*Δ*68* cells (S2 Table) and or inactivation of these enzymes due to the absence of iron sulfur clusters in *iscUC63F* cells. As ROS levels are notoriously difficult to determine directly [16], we turned to the OxyR regulon that is induced by H_2_O_2_. We did not observe major changes in expression of the OxyR regulated *katG* gene in *iscUC63F* and *fre*Δ*68* cells (S6 Fig) which could indicate that the cytoplasmic level of H_2_0_2_ is not reduced in the mutants. However, the observation that *katG* expression is not reduced during anaerobic growth either [73], suggest that *katG* is not a good reporter for H_2_O_2_ levels close to or especially below wild-type level. Our observation that *katG* expression was reduced when ArcA was overproduced could be independent of H_2_O_2_ and result in repression of *rpoS* which in turn resulted in production of less KatG [74]

Although we have not been able to measure a reduction in the cellular ROS level, we favor a model in which *iscUC63F*, *fre*Δ*68* and *atpAB* mutants sustain growth of overinitiating cells by reducing ROS and hence oxidative damage to the DNA. This is supported by the observation that deletion of *sodB* which presumably result in accumulation of superoxide, is deleterious for overinitiating cells, but tolerated by wild-type cells. Overall we suggest that *iscUC63F*, *fre*Δ*68* and *atpAB* mutations are just a few of many putative RIDA suppressors that affect the cellular redox balance and ultimately chromosome stability. We do however recognize that this is a model only, and that *iscUC63F*, *fre*Δ*68* and *atpAB* mutations have pleiotropic effects on cellular physiology and that mechanisms not involving ROS reduction may at least in part be involved.

RIDA deficient cells carrying the *iscUC63F*, *fre*Δ*68* and *atpAB* suppressor mutations grow relatively poorly compared to other suppressors that downregulate initiation of replication (i.e. affecting DnaA, SeqA; [23]). This most likely reflects the partial suppression of DNA damages, but a too high DNA concentration could *per se* be a challenge for the cells in relation to processes such as supercoiling, repair and segregation. This may explain the slow growth of this type of suppressors relative to those that affect the initiation of replication. This also stresses the fact that *E*. *coli* has evolved a multitude of mechanisms that ensure that the origin concentration remains relatively invariant regardless of the growth conditions [2].

## Materials and Methods

### Growth conditions

Cells were grown in Luria–Bertani (LB) medium or AB minimal medium [75] supplemented with 0.2% or 0.04% glucose, 0.5% casamino acids and 10 μg/ml thiamine. LB with 0.2% glucose medium was used for anaerobic growth. Unless specified, all cells were cultured at 37°C. When necessary, antibiotic selection was maintained at the following concentrations: kanamycin 50 μg/ml; chloramphenicol, 20 μg/ml; ampicillin, 150 μg/ml. Cell growth was monitored by measuring optical density at 450 nm for AB minimal medium. Anaerobic growth was performed in an anaerobic jar using anaerobic atmosphere generation bags (BD).

### Bacterial strains and plasmids

All strains used are derivatives of MG1655 (F-λ-rph-1) [76]. The *hda* deletion described previously [26] was moved by P1 mediated generalized transduction [77].

Mutations from the KEIO collection [78] were moved by P1 transduction into MG1655 using lysates of: JW4364 (Δ*arcA*), JW0723-2 (Δ*cydB*), JW3712-1 (Δ*atpA*), JW3716-1 (Δ*atpB*), JW2513 (Δ*iscU*) JW3879 (Δ*sodA*), JW1648 (Δ*sodB*).

The *katG*::*lacZ* gene fusion from strain AL441 [51] was moved into MG1655, *iscUC63F* and *fre*Δ*68* by P1 transduction.

The *cyd* operon and *arcA* gene were amplified from strain MG1655 using primers pairs 5’-CTCTAGATTAAGGAGGCCATATGTTAGATATAGTCGAACT-3’/ 5’-AGAGAATTCTGATTTAAAAGAA-3’ and 5’-CTCTAGATTAAGGAGGCCATATGCAGACCCCGCACATTCT-3’/ 5’-CGAATTCTTAATCTTCCAGATCACCGC-3’ respectively. The PCR products were digested with XbaI / EcoRI and cloned into plasmid pFH2102 digested with the same enzymes resulting in plasmids pFH2102CYDOP and pFH2102ARCA. The *cyd* operon and the *arcA* gene were then amplified from pFH2102CYDOP and pFH2102ARCA using a primer annealing upstream of the Shine Dalgarno GTTGACTTGTGAGCGGATAA and 5’-AGAGAATTCTGATTTAAAAGAA-3’ (for cyd) or 5’-CGAATTCTTAATCTTCCAGATCACCGC-3’ (for *arcA*). The PCR products were digested with XhoI/EcoRI and cloned into plasmid pNDM220 digested with the same enzymes, resulting in plasmids pNDM-*cyd* and pNDM-*arcA*

### Microarray analysis

Strains were grown exponentially in ABTG supplemented with 0.5% casamino acids. At an optical density OD_450_ = 0.3, 35 ml of culture was transferred to a cold tube containing 5 ml frozen water and centrifugated at 4°C at 10000g for 5min. Pellets were resuspended in 0.5ml ice cold TE-Buffer, transferred to an Eppendorph tube containing 250μl lysis buffer (2% SDS, 16mM EDTA and 200mM NaCl) and 750μl Phenol, whirly mixed and placed at 65°C for 10min with whirly mixing every 3–4 min. Following phenol extraction, each sample was treated with Dnase I for one hour at 4°C and an RNA clean up was made using the RNeasy Mini Spin Column kit from QIAGEN.

cDNA was synthesized using the Revert Aid H Minus first strand synthesis Kit from Thermo Scientific. The cDNA was fragmented with DNAse I in One-Phor-All buffer (Amersham Biosciences) and end-labeled with Biotin using the Enzo BioArray Terminal Labeling Kit. The fluorescent labeled cDNA hybrizized to GeneChip® E. coli Genome 2.0 Array was scanned as described in the Affymetrix UserGuide (www.affymetrix.com) and analyzed using GeneChip Analysis Suite software. Following Robust Multi-array Average (RMA) normalization, a cut off for low expression was applied and genes whose expression varied in the wt strains excluded from the analysis. Raw RMA normalization data can be found in S1 Appendix.

### Determination of ori/ter ratio

Was done by quantitative PCR was performed as previously described [14] with modifications. One milliliter of exponentially growing cells (OD_450_ ~ 0.2) is harvested and put on ice, centrifuged 5 minute 8000 g, the supernatant discarded and cells resuspended in 100 μl of cold 10 mM Tris pH7.5 and fixed by adding 1 ml of 77% ethanol. The samples were stored at 4°C. For qPCR analysis, 100 μl of ethanol fixed cells were centrifuged 7 minutes at 17000 g, the supernatant discarded and the samples were centrifuged for another 30 seconds at 17000 g and the remaining ethanol removed. The cell pellet was resuspended in 1ml cold water and 2 μl of the cell suspension was used as template for qPCR analysis. The Quantitative-PCR was performed using Takara SYBR Premix Ex Taq II (RR820A) in a BioRAD CFX96. All *ori*/*ter* ratios were normalized to the *ori*/*ter* ratio of MG1655 treated with rifampicin for 2h. The origin and terminus were quantified using primers 5′-TTCGATCACCCCTGCGTACA-3′ and 5′-CGCAACAGCATGGCGATAAC-3′ for the origin and 5′-TTGAGCTGCGCCTCATCAAG-3′ and 5′-TCAACGTGCGAGCGATGAAT-3′ for terminus as previously reported [26].

### Flow cytometry

Flow cytometry was performed as described previously [79] using an Apogee A10 Bryte instrument. For each sample, 30 000 to 200 000 cells were analyzed. Numbers of origins per cell and relative cell mass were determined as described previously [79].

### Extraction and quantification of intracellular cofactors

Intracellular ATP and ADP were extracted as previously described [80]. ATP/ADP ratios were measured using a luciferin-luciferase ATP kit (Microbial ATP Kit HS, BioThema AB, Sweden). Intracellular NADH and NAD^+^ was extracted as previously described [81]. The NADH/NAD^+^ ratio was quantified by a luciferase assay provided by the kit NAD^+^/NADH-Glo Assay (Promega). The luminescence from each assay was measured using the Infinite® M1000 PRO microplate reader (TECAN) with the 96-well microplates from Greiner Bio-one (Cat. No. 655901).

### Quantification of glucose and fermentation products

High-performance liquid chromatography (HPLC) was used to measure the concentration of glucose, acetate, formate, ethanol, succinate, citrate and lactate as previously described [81]

Quantification of oxygen consumption was made in a Bioreactor (Sartorius Biostat Q, 500 mL working volume) equipped with an O_2_ electrode (Mettler Toledo, Switzerland).

### Pulsed-field gel electrophoresis

Was done as described previously [14]

### RT-qPCR

RT qPCR was performed on phenol extracted total RNA using QuantiNova SYBR Green RT-PCR Kit using supplier recommendations and specific primers for *cydA* (5’-TGCGGCCTGTATACCCTGTTCC-3’ and 5’-CGTGCCGGCTGAGTAGTCGTG-3’), *cyoA* (5’-CCGCTGGCACACGACGAGA-3’ and 5’-AAGCGATTTCATTCACGGTAGCA-3’) and *sdhD*(5’-GATCGGTTTCTTCGCCTCTG-3’ and 5’-CGGTCAACACCTGCCACAT-3’) [82] and normalized to *rpoA* (5’-TTGATATCGAGCAAGTGAGTTCG-3’ and 5’- GCATCGATGAGAGCAGAATACG-3’) [27].

## Supporting Information

S1 Table Genes with altered expression in *IscUC63F*. Gene expression is represented as ratio relative to MG1655. Fold changes for genes that are repressed are expressed in negative values. All genes in a given operon where a relevantly expressed gene is located are shown; the gene whose expression is most affected in the operon is highlighted in red (repressed) or green (overexpressed).
(DOCX)

S2 Table Genes displaying Fre dependent change in expression in *fre*.Δ*68* and Δ*fre*. Results from Fre overproduction are also shown (pFRE). Gene expression is represented as ratio relative to MG1655. Fold changes for genes that are repressed are expressed in negative values. All genes in a given operon where a relevantly expressed gene is located are shown; the gene whose expression is most affected in the operon is highlighted in red (repressed) or green (overexpressed). Genes coding for dehydrogenases are highlighted in yellow Genes coding for cytochrome terminal oxidase are highlighted in blue. Gene products inhibited by NADH are followed by an asterisk. (1) Operons or genes that are differentially expressed in a Δ*arcA* strain compared to wild-type [25]. (2) Operons or genes directly regulated by ArcA [25]. (3) Operons or genes that are differentially expressed in a Δ*arcA* strain compared to wild-type [24].
(DOCX)

S3 Table Level of *cydA*, *sdhD* and *cyoA* expression measured by Quantitative PCR. The indicated cells were grown in AB minimal medium supplemented with 0.2% glucose and 0.5% casamino acids. Relative abundance of *cydA*, *sdhD* and *cyoA* mRNA relative to *rpoA* mRNA was measured by quantitative RT-PCR. Shown is the mean ± s.d. (n = 3). For comparison, values for the same genes derived from the microarray analysis are included.
(DOCX)

S1 Fig Visualization of strand breaks by PFGE. Cells were grown aerobically in AB minimal medium supplemented with 0.2% glucose and 0.5% casamino acids except *hda* cells that were grown anaerobically and shifted for 4 hours to aerobic conditions. Wild-type cells treated for 30 minutes with 2μg/ml ciprofloxacin are used as strand break control. Top: PFGE gel. Bottom: line scan analysis of relevant lanes.
(PDF)

S2 Fig Hda is dispensable during slow growth. A) The *hda*::*cat* mutation was introduced into wild-type cells under anaerobic conditions, restreaked on AB minimal medium plates supplemented with 0.2% glycerol along with wild-type and *hda iscUC63F* cells and incubated aerobically. B) The *hda*::*cat* mutation was introduced into wild-type cells under anaerobic conditions, restreaked on AB minimal medium plates supplemented with 0.2% glucose and 0.5% casamino acids along with wild-type and *hda iscUC63F* cells and incubated aerobically. C) *Hda* clones obtained on AB minimal medium supplemented with 0.2% glycerol were restreaked on LB plates along with wild-type and *hda iscUC63F* cells and incubated aerobically. D) Cells were grown exponentially in AB minimal medium supplemented with 0.2% glycerol and treated with rifampicin and cephalexin prior to flow cytometric analysis. E) Cells were grown exponentially in AB minimal medium then shifted to AB minimal medium supplemented with 0.2% glucose and 0.5% casamino acids medium for 3 mass doubling time or F) LB medium for 3 mass doubling time and treated with rifampicin and cephalexin prior to flow cytometric analysis. Each panel represents a minimum of 30000 cells. The average *ori*/cell (O/C), *ori*/mass (O/M) relative to wild-type and mass doubling time (τ) are inserted in the histograms.
(PDF)

S3 Fig Cell cycle profile of *iscU* and *fre* mutants grown in LB. Cells were grown exponentially in LB medium and treated with rifampicin and cephalexin prior to flow cytometric analysis. Each panel represents a minimum of 30000 cells. The average *ori*/cell (O/C), *ori*/mass (O/M) relative to wild-type and mass doubling time (τ) are inserted in the histograms.
(PDF)

S4 Fig Cytochrome bd-1 function is required for *atpA*, *atpB and iscUC63F* survival in absence of *hda*. The *hda*::*cat* mutation was introduced into the indicated strains under anaerobic conditions, restreaked on LB agar and incubated aerobically.
(PDF)

S5 Fig The OxyR and SoxRS regulons in *iscU* and *fre* mutants. The expression level of selected OxyR and SoxRS controlled genes relative to wild-type extracted from the microarray experiment.
(PDF)

S6 Fig *katG* expression in *iscUC63F* and *fre*Δ*68*, Δ*cydB* and ArcA overproducing strains. Expression of *katG*::*lacZ* in cells growing in LB medium was measured by β-galactosidase assay. The results are expressed relative to wild-type.
(PDF)

S7 Fig Effect of *sodA* and *sodB* mutations on growth of *hda fre*Δ*68* cells. The *sodA*::*kan* and *sodB*::*kan* mutations were introduced into *hda fre*Δ*68* cells under anaerobic conditions and restreaked under aerobic conditions on LB agar.
(PDF)

S1 Appendix RMA normalized transcriptomic data. (XLSX)
