# Supplementary material for: Re-wiring of energy metabolism promotes viability during hyperreplication stress in E. coli
Source: PLoS Genet. 2017 Jan 27;13(1):e1006590. doi: 10.1371/journal.pgen.1006590 (PMC5302844; doi:10.1371/journal.pgen.1006590)
Supplement: S1 Table — Gene expression is represented as ratio relative to MG1655. Fold changes for genes that are repressed are expressed in negative values. All genes in a given operon where a relevantly expressed gene is located are shown; the gene whose expression is most affected in the operon is highlighted in red (repressed) or green (overexpressed). (DOCX) [file pgen.1006590.s001.docx]

| **Genes name** | **Ratio *iscUC63F*** **vs wt** | **Fe-S cluster** |
| --- | --- | --- |
| **Fe-S cluster assembly protein** | | |
| *sufE* | **2.3** |  |
| *sufS* | **2.4** |  |
| *sufD* | **2.2** |  |
| *sufC* | **2.4** |  |
| *sufB* | **3.0** | Fe-S |
| *sufA* | **3.2** | Fe-S |
| *iscR* | 1.6 | Fe-S |
| *iscS* | 1.7 |  |
| *iscU* | **1.7** | Fe-S |
| *iscA* | **1.8** | Fe-S |
| *hscB* | **2.0** |  |
| *hscA* | 1.2 |  |
| *fdx* | 1.4 | Fe-S |
| *iscx* | 1.4 |  |
| **TCA cycle/ Respiration** | | |
| *gltA* | **-1.8** |  |
| *sdhC* | **-2.5** |  |
| *sdhD* | **-2.3** |  |
| *sdhA* | **-2.1** |  |
| *sdhB* | **-2.1** | Fe-S |
| *b0725* | -1.4 |  |
| *sucA* | -1.7 |  |
| *sucB* | -1.6 |  |
| *sucC* | **-1.7** |  |
| *sucD* | -1.4 |  |
| *acnA* | **-2.0** | Fe-S |
| *nuoN* | -1.2 |  |
| *nuoM* | -1.3 |  |
| *nuoL* | -1.3 |  |
| *nuoK* | -1.4 |  |
| *nuoJ* | -1.2 |  |
| *nuoI* | -1.4 | Fe-S |
| *nuoH* | -1.4 |  |
| *nuoG* | -1.4 | Fe-S |
| *nuoF* | -1.2 | Fe-S |
| *nuoE* | -1.5 | Fe-S |
| *nuoC* | -1.4 |  |
| *nuoB* | -1.4 | Fe-S |
| *nuoA* | **-1.7** |  |
| *fdhE* | -1.7 |  |
| *fdoI* | **-2.8** |  |
| *fdoH* | **-3.1** | Fe-S |
| *fdoG* | **-3.4** | Fe-S |
| *ndh* | **1.9** |  |
| **Central intermediary metabolism** | | |
| *ppsR* | **2.3** |  |
| *ackA* | **2.0** |  |
| *ldhA* | **1.8** |  |
| *dhaR* | **-1.8** |  |
| *glgS* | **-2.1** |  |
| *glk* | **-1.9** |  |
| *talA* | -1.4 |  |
| *tktB* | **-1.8** |  |
| *mhpR* | **-2.0** |  |
| *nagC* | -1.4 |  |
| *nagA* | **-1.8** |  |
| *nagB* | -1.3 |  |
| *glcC* | **-1.8** |  |
| *mtlD* | **-1.8** |  |
| *mtlR* | -1.4 |  |
| *can* | **2.3** |  |
| **DNA Replication/nucleotide synthesis** | | |
| *sbmC* | **-2.0** |  |
| *folA* | **-1.8** |  |
| *nrdH* | **2.1** |  |
| *nrdI* | **1.8** |  |
| *nrdE* | 1.5 |  |
| *nrdF* | **2.3** |  |
| **Phosphate homeostasis** | | |
| *phnE_1* | **-3.6** |  |
| *phnE_2* | -1.5 |  |
| *phnD* | **-4.0** |  |
| *phnC* | **-2.8** |  |
| *phnA* | **-2.1** |  |
| *phoQ* | **-2.0** |  |
| *phoP* | **-1.8** |  |
| *phoA* | **-2.3** |  |
| *psiF* | **-1.8** |  |
| *phoE* | **-1.7** |  |
| *ugpC* | **-1.8** |  |
| *ugpE* | **-2.0** |  |
| *ugpA* | **-3.2** |  |
| **Oxidative stress** | | |
| *sodB* | **-2.5** |  |
| *yhcN* | **-2.2** |  |
| *bcp* | **1.7** |  |
| *hmpA* | **2.5** |  |
| **Iron homeostasis** | | |
| *nmpC* | **1.7** |  |
| *EfeU_1* | **2.1** |  |
| *EfeU_2* | 1.5 |  |
| *hemA* | **-2.0** |  |
| *prfA* | -1.6 |  |
| *prmC* | **-1.8** |  |
| *ftn* | **-2.5** |  |
| *metK* | **1.8** |  |
| **Biotin biosynthesis** | | |
| *bioA* | **2.2** |  |
| *bioB* | **3.1** | Fe-S |
| *bioF* | **2.8** |  |
| *bioD* | **2.6** |  |
| *mioC* | 1.6 |  |
| **Amino acid biosynthesis** | | |
| *hisG* | -1.4 |  |
| *hisD* | **-1.7** |  |
| *hisC* | **-1.7** |  |
| *hisB* | **-1.9** |  |
| *hisH* | **-1.7** |  |
| *hisA* | -1.6 |  |
| *hisF* | -1.3 |  |
| *hisI* | -1.2 |  |
| *trpA* | -1.5 |  |
| *trpB* | -1.5 |  |
| *trpC* | **-1.7** |  |
| *trpD* | **-1.9** |  |
| *trpE* | **-1.8** |  |
| *trpL* | -1.3 |  |
| *astE* | -1.2 |  |
| *astB* | -1.3 |  |
| *astD* | **-3.7** |  |
| *astC* | **-2.2** |  |
| *yffB* | **-2.0** |  |
| *dapE* | **-1.9** |  |
| *cysH* | -1.4 |  |
| *cysI* | -1.4 | Fe-S |
| *cysJ* | **-2.4** |  |
| *cysC* | -1.3 |  |
| *cysN* | -1.3 |  |
| *cysD* | **-1.7** |  |
| *cysA* | **-1.8** |  |
| *cysW* | -1.3 |  |
| *cysU* | -1.6 |  |
| *cysP* | -1.5 |  |
| *ilvG_1* | -1.5 |  |
| *ilvG_2* | **-1.7** |  |
| *ilvM* | **-2.0** |  |
| *ilvE* | -1.6 |  |
| *ilvD* | -1.3 | Fe-S |
| *ilvA* | -1.1 |  |
| *proV* | **2.3** |  |
| *proW* | **2.4** |  |
| *proX* | **2.8** |  |
| *asnB* | **1.7** |  |
| **Putrescein biosynthesis** | | |
| *puuD* | **-2.0** |  |
| *puuR* | **-1.9** |  |
| *puuC* | **-1.9** |  |
| *puuB* | -1.5 |  |
| *puuE* | -1.5 |  |
| **Stress response** | | |
| *ibpB* | **2.6** |  |
| *ibpA* | **2.1** |  |
| *UspG* | **-2.9** |  |
| *cbpA* | **-2.2** |  |
| *cbpM* | **-1.8** |  |
| **Acid resistance** | | |
| *gadB* | **-3.3** |  |
| *gadC* | **-2.9** |  |
| *gadA* | **-5.6** |  |
| *yhiD* | **-1.8** |  |
| *hdeB* | -1.3 |  |
| *hdeA* | -1.4 |  |
| *hdeD* | **-1.7** |  |
| *clcA* | **-1.7** |  |
| *ybaS* | **-3.4** |  |
| *ybaT* | **-1.8** |  |
| *cueR* | **-1.9** |  |
| **tellurite resistance** | | |
| *tehB* | **2.2** |  |
| *tehA* | **2.2** |  |
| **Multidrug resistance** | | |
| *mdtG* | **-1.8** |  |
| *mdfA* | **-1.7** |  |
| *mprA* | **1.8** |  |
| **Attachment /motility** | | |
| *slyA* | **1.9** |  |
| *flgN* | **1.8** |  |
| *flgM* | 1.5 |  |
| *(flgA)* | **2.1** |  |
| *(flgB)* | **5.7** |  |
| *(flgC)* | **3.3** |  |
| *(flgD)* | **3.5** |  |
| *(flgE)* | **7.2** |  |
| *flgF* | 1.4 |  |
| *(flgG)* | **2.4** |  |
| *(flgH)* | 1.6 |  |
| *(flgI)* | 1.2 |  |
| *(flgJ)* | 1.4 |  |
| *(flgK)* | 1.6 |  |
| *flgL* | **1.8** |  |
| *fimA* | **-4.8** |  |
| *fimC* | **-2.6** |  |
| *fimD* | **-2.1** |  |
| *fimF* | -1.6 |  |
| *fimG* | -1.6 |  |
| *fimH* | **-1.9** |  |
| *lrhA* | **-2.5** |  |
| *ydeQ* | **-2.0** |  |
| *ydeR* | **-2.7** |  |
| *ydeS* | **-2.3** |  |
| *ydeT* | **-2.7** |  |
| *gmr* | **-2.2** |  |
| **Translation** | | |
| *rbbA* | **-2.3** |  |
| *yhiL* | **-2.3** |  |
| **Cell wall /membrane biogenesis** | | |
| *rfaS* | **1.8** |  |
| *dacC* | **-1.9** |  |
| *ybjG* | **-1.9** |  |
| *arnC* | **-1.9** |  |
| *ept* | **-1.7** |  |
| **Non-coding RNA** | | |
| *rtT* | -1.6 |  |
| *tpr* | **-1.7** |  |
| **Unknown function** | | |
| *ygbA* | **1.8** |  |
| *YeeN* | **2.0** |  |
| *yceA* | **2.0** |  |
| *ybiV* | **1.7** |  |
| *ybaP* | **-1.9** |  |
| *yahM* | **-2.8** |  |
| *ydhI* | **-1.8** |  |
| *ydhJ* | **-1.9** |  |
| *ydhI* | -1.6 |  |
| *yghG* | **-1.8** |  |
| *yghJ* | **-1.8** |  |
| *yhhY* | **-1.8** |  |
| *ybhP* | **-1.8** |  |
| *yfhL* | **-1.7** | Fe-S |
| *yieE* | **-1.7** |  |
| *csiD* | **-1.7** |  |
| *ybaY* | **-1.7** |  |
| *ykgA* | **-1.7** |  |

**S1 Table. Genes with altered expression in *IscUC63F***. Gene expression is represented as ratio relative to wt MG1655. Fold changes for genes that are repressed are expressed in negative values. All genes in a given operon where a relevantly expressed gene is located are shown; the gene whose expression is most affected in the operon is highlighted in red (repressed) or green (overexpressed).
