# Supplementary material for: Re-wiring of energy metabolism promotes viability during hyperreplication stress in E. coli
Source: PLoS Genet. 2017 Jan 27;13(1):e1006590. doi: 10.1371/journal.pgen.1006590 (PMC5302844; doi:10.1371/journal.pgen.1006590)
Supplement: S2 Table — Gene expression is represented as ratio relative to MG1655. Fold changes for genes that are repressed are expressed in negative values. All genes in a given operon where a relevantly expressed gene is located are shown; the gene whose expression is most affected in the operon is highlighted in red (repressed) or green (overexpressed). Genes coding for dehydrogenases are highlighted in yellow Genes coding for cytochrome terminal oxidase are highlighted in blue. Gene products inhibited by NADH are followed by an asterisk. (1) Operons or genes that are differentially expressed in a ΔarcA strain compared to wild-type [25]. (2) Operons or genes directly regulated by ArcA [25]. (3) Operons or genes that are differentially expressed in a ΔarcA strain compared to wild-type [24]. (DOCX) [file pgen.1006590.s002.docx]

| **Genes name** | ***fre*Δ68  vs wt** | ** Δ*fre* vs wt** | **pFRE vs wt** | **ArcA Regulon** |
| --- | --- | --- | --- | --- |
| **TCA/ Respiration/Redox homeostasis** | | | |  |
| ***fre*** | **-1.6** | **-4.3** | **14.3** |  |
| *cyoE* | **-4.3** | **-5.0** | -1.0 | (1).(2) |
| *cyoD* | **-4.3** | **-4.2** | 1.0 | (1).(2) |
| *cyoC* | **-4.0** | **-3.8** | 1.1 | (1).(2) |
| *cyoB* | **-3.7** | **-3.4** | 1.1 | (1).(2) |
| *cyoA* | **-3.2** | **-3.3** | 1.1 | (1).(2) |
| *gltA* ******* | **-3.0** | **-4.2** | -1.1 | (1).(2).(3) |
| *sdhC* | **-7.7** | **-9.1** | 1.1 | (1).(2).(3) |
| *sdhD* | **-6.3** | **-7.1** | 1.1 | (1).(2).(3) |
| *sdhA* | **-5.0** | **-5.6** | 1.2 | (1).(2).(3) |
| *sdhB* | **-5.6** | **-6.3** | 1.1 | (1).(2).(3) |
| b0725 | **-3.1** | **-3.6** | 1.1 | (1).(2) |
| *sucA* | **-3.1** | **-3.3** | 1.2 | (1).(2) |
| *sucB* | **-2.6** | **-2.5** | 1.2 | (1).(2) |
| *sucC* | **-2.8** | **-2.6** | 1.2 | (1).(2) |
| *sucD* | **-2.3** | **-2.3** | 1.3 | (1).(2) |
| *nuoN* | -1.6 | -1.4 | 1.1 | (1).(2) |
| *nuoM* | **-1.8** | **-1.5** | 1.0 | (1).(2) |
| *nuoL* | **-1.8** | **-1.5** | 1.2 | (1).(2) |
| *nuoK* | **-2.1** | **-1.6** | 1.2 | (1).(2) |
| *nuoJ* | **-1.8** | -1.4 | 1.2 | (1).(2) |
| *nuoI* | **-2.3** | **-1.7** | 1.1 | (1).(2) |
| *nuoH* | **-2.0** | **-1.5** | 1.2 | (1).(2) |
| *nuoG* | **-2.4** | **-1.9** | 1.2 | (1).(2) |
| *nuoF* | -1.3 | -1.1 | 1.1 | (1).(2) |
| *nuoE* | **-2.6** | **-1.7** | 1.1 | (1).(2) |
| *nuoC* | **-2.3** | **-1.6** | 1.1 | (1).(2) |
| *nuoB* | **-2.3** | **-1.8** | 1.0 | (1).(2) |
| *nuoA* | **-2.5** | **-2.1** | -1.1 | (1).(2) |
| *fdoI* | **-2.1** | **-2.0** | 1.1 | (1).(2) |
|  |  |  |  |  |
| *fdoH* | **-2.1** | **-2.0** | 1.1 | (1).(2) |
| *fdoG* | **-2.0** | **-2.1** | 1.1 | (1).(2) |
| *fdhE* | -1.4 | -1.3 | 1.0 | (1).(2) |
| *SthA(udhA)* | **-5.9** | **-6.3** | 1.0 | (1).(2) |
| *fabR* | -1.3 | **-1.6** | -1.3 |  |
| *yijD* | -1.3 | **-1.9** | -1.4 |  |
| *acnA* | **-3.3** | **-3.6** | 1.0 | (1) |
| *acnB* | **-3.8** | **-3.6** | 1.2 | (1).(2) |
| *mqo* | **-2.9** | **-2.6** | 1.3 | (1).(2) |
| *fumC* | **-2.6** | **-2.9** | -1.1 | (1).(2) |
| *fumA* | **-3.4** | **-3.0** | 1.2 | (1).(2) |
| *aceB* | -1.3 | **-1.5** | -1.2 | (1) |
| *aceA* | **-1.5** | **-1.8** | -1.2 | (1) |
| *aceK* | **-1.4** | **-1.8** | -1.1 | (1) |
| *mdh* | **-2.4** | **-3.1** | -1.2 | (1).(2) .(3) |
| *icdA* | **-2.2** | **-2.6** | 1.1 | (1).(2) .(3) |
| *nudE* | **-1.7** | **-2.0** | -1.1 | (1).(2) |
| *trxC* | **-1.7** | **-1.8** | -1.0 | (1).(2) |
| *yfiP* | **-1.5** | -1.2 | -1.1 |  |
| *cydA* | **6.4** | **6.1** | -1.0 | (3) |
| *cydB* | **6.0** | **6.0** | -1.0 | (3) |
| *ybgE* | **3.9** | **3.1** | -1.1 |  |
| *AppC* | **2.0** | 1.5 | -1.4 | (1) |
| *AppB* | 1.5 | 1.2 | -1.2 | (1) |
| *AppY* | **3.4** | **2.2** | -1.2 | (1).(2) |
| *ndh* | **1.9** | **2.2** | 1.4 | (3) |
| *nadB* | 1.3 | **2.5** | -1.1 | (1) |
| **Intermediary carbon Metabolism** | | | |  |
| *gcd* | **-4.5** | **-5.0** | -1.0 | (1).(2) |
| *aldA* ******* | **-2.1** | **-2.2** | -1.1 | (1).(2) .(3) |
| *glcC* | **-2.2** | **-2.1** | -1.1 | (1).(2) .(3) |
| *glk* | **-1.7** | **-1.5** | -1.2 |  |
| *lldP* | **-1.9** | **-1.8** | -1.1 | (1).(2) .(3) |
| *lldR* | **-3.3** | **-3.4** | -1.2 | (1).(2) .(3) |
| *lldD* | -1.4 | -1.5 | -1.0 | (1).(2) .(3) |
| *focA* | **2.1** | **1.9** | -1.1 | (3) |
| *ackA* | 1.5 | **2.0** | 1.5 |  |
| *gatD* | **1.8** | **1.9** | -1.1 |  |
| *gatC* | 1.5 | 1.6 | -1.0 |  |
| *gatB* | **2.1** | **2.4** | 1.1 |  |
| *gatA* | **2.0** | **2.1** | -1.1 |  |
| *gatZ* | 1.6 | **1.8** | -1.1 |  |
| *gatY* | 1.4 | 1.7 | -1.1 |  |
| *manX* | **1.8** | **1.8** | 1.1 | (3) |
| *manY* | 1.7 | 1.5 | 1.1 |  |
| *manZ* | 1.6 | 1.5 | 1.0 |  |
| *kgtP* | **-2.8** | **-2.9** | -1.1 | (1).(2).(3) |
| *ppsR* | **1.7** | **1.8** | 1.1 |  |
| **DNA Replication/nucleotide synthesis** | | | |  |
| *holE* | **1.9** | 1.7 | 1.0 |  |
| *cnu* | **1.9** | 1.4 | -1.4 | (1) |
| *nepI* | **-1.5** | **-1.8** | -1.0 | (1).(2) |
| *ndk* | **-2.5** | **-2.5** | 1.2 | (1).(2) .(3) |
| *folA* | **-1.7** | **-1.9** | -1.3 |  |
| **Phosphate Homeostasis** | | | |  |
| *phoQ* | **-1.9** | **-1.9** | -1.3 |  |
| *phoP* | **-1.6** | **-1.8** | -1.2 |  |
| **Oxidative stress** | | | |  |
| *sodA* | -1.6 | -1.5 | 1.0 | (3) |
| *yeaC* | **-2.4** | **-2.8** | -1.1 | (1) |
| *msrB* | **-2.4** | **-2.8** | 1.1 | (1) |
| *yhcN* | **-1.6** | **-3.1** | -1.3 |  |
| *kefB* | **-2.8** | **-2.6** | -1.3 | (1).(2).(3) |
| *kefG* | **-2.2** | **-2.4** | -1.2 | (1).(2).(3) |
| **Iron homeostasis** | | | |  |
| *feoA* | **2.3** | **2.2** | -1.1 | (1).(2).(3) |
| *feoB* | **1.9** | 1.7 | -1.1 | (1).(2) |
| *feoC* | **1.9** | 1.7 | -1.1 | (1).(2) |
| *nmpC* | **3.4** | **3.8** | 1.4 |  |
| *cirA* | 1.1 | 1.5 | **1.9** | (1).(2) |
| *(ftn)* | **-1.6** | **-1.7** | **-1.5** |  |
| *efeU_1* | -1.3 | **-1.6** | 1.2 | (1) |
| *efeU_2* | **-1.6** | **-1.7** | 1.2 | (1) |
| *efeO* | **-1.6** | -1.4 | 1.2 |  |
| *efeB* | **-1.6** | -1.4 | 1.1 |  |
| *fiu* | **-1.6** | -1.1 | **2.0** |  |
| *yncD* | **-2.5** | **-3.1** | 1.0 | (1).(2) |
| **Amino Acids biosynthesis** | | | |  |
| *aspA* | **1.8** | 1.4 | -1.1 | (1) |
| *dcuA* | 1.5 | 1.5 | 1.0 |  |
| *hisP* | **-1.7** | **-1.5** | -1.0 | (1).(2) |
|  |  |  |  |  |
| *hisM* | **-1.9** | **-2.1** | -1.1 | (1).(2) |
| *hisQ* | **-1.7** | **-1.7** | -1.1 | (1).(2) |
| *hisJ* | **-1.9** | **-1.9** | -1.0 | (1).(2) |
| *argT* | **-3.6** | **-5.3** | -1.1 | (1).(2) |
| *dadA* | **-2.9** | **-2.3** | -1.1 | (1) |
| *dadX* | **-3.2** | **-2.4** | -1.2 | (1) |
| *cvrA* | **-1.7** | -1.2 | -1.1 |  |
| *astE* | -1.2 | -1.1 | 1.0 | (1).(2) |
| *astB* | -1.4 | -1.1 | 1.2 | (1).(2) |
| *astD* | **-4.5** | **-5.0** | -1.1 | (1).(2) |
| *astC* | **-2.8** | **-2.9** | -1.0 | (1).(2) |
| *yffB* | **-1.5** | **-2.0** | -1.2 |  |
| *dapE* | -1.4 | **-1.6** | -1.2 |  |
| *putA* | **-2.9** | **-2.7** | 1.1 | (1).(2) |
| **Glycine /Betain/ Spermidine / putrescein biosynthesis** | | | |  |
| *betA* | **-2.2** | **-1.8** | 1.1 | (1).(2) |
| *betB* | **-1.9** | **-1.7** | 1.2 | (1).(2) |
| *betI* | **-2.7** | **-2.3** | 1.1 | (1).(2) |
| *betT* | **-1.7** | **-1.7** | 1.1 | (1).(2) |
| *mhpR* | **-2.9** | **-2.6** | 1.0 | (1).(2) |
| *proP* | **-1.7** | **-1.7** | -1.1 |  |
| *puuD* | **-2.6** | **-2.9** | -1.1 | (1).(2) |
| *puuR* | **-2.5** | **-2.6** | -1.1 | (1).(2) |
| *puuC* | **-2.5** | **-1.8** | 1.1 | (1) |
| *puuB* | -1.4 | -1.4 | 1.1 | (1) |
| *puuE* | -1.2 | -1.4 | 1.0 | (1) |
| *potF* | **-2.1** | **-2.2** | -1.1 | (1).(2) |
| *potG* | **-2.9** | **-2.6** | -1.1 | (1).(2) |
| *potH* | -1.1 | -1.1 | 1.0 | (1).(2) |
| *potI* | -1.4 | -1.1 | 1.0 | (1).(2) |
| *cysM* | 1.1 | -1.1 | -1.1 | (3) |
| *cysA* | -1.3 | -1.6 | -1.4 |  |
| *cysW* | -1.3 | -1.4 | -1.3 |  |
| *cysU* | -1.4 | -1.4 | -1.4 |  |
| *cysP* | **-1.7** | -1.4 | -1.3 |  |
| *cysH* | -1.1 | **-1.7** | -1.2 |  |
| *cysI* | -1.2 | -1.4 | -1.1 |  |
| *cysJ* | **-1.5** | **-1.6** | -1.3 |  |
| **Acid resistance** | | | |  |
| *gadB* | **1.9** | **2.7** | **-2.1** | (1) |
| *gadC* | **1.8** | **2.5** | **-2.0** | (1) |
| *gadA* | 1.5 | **2.3** | **-2.3** | (1) |
| *gadX* | **2.6** | **2.1** | -1.1 | (1).(2) |
| *slp* | **1.9** | 1.6 | **-1.5** | (1).(2) |
| *dctR* | **2.0** | **2.0** | **-1.7** | (1).(2) |
| *yhiD* | **2.7** | **2.4** | **-1.8** | (1) |
| *hdeB* | **1.8** | **1.8** | **-1.6** | (1) |
| *hdeA* | 1.7 | 1.7 | **-1.6** | (1) |
| *hdeD* | **2.4** | **2.3** | **-1.7** | (1) |
| *gadE* | **3.1** | **3.2** | **-1.9** | (1).(2) |
| **Multidrug resistance** | | | |  |
| *mdtE* | **1.9** | **2.5** | -1.0 | (1).(2) |
| *mdtF* | 1.7 | **2.4** | -1.1 | (1).(2) |
| **Heat chock resistance** | | | |  |
| *ibpB* | **1.8** | **1.8** | 1.2 |  |
| *ibpA* | **1.8** | 1.5 | 1.2 |  |
| *htpG* | **1.8** | 1.4 | 1.1 |  |
| **Osmotic chock resistance** | | | |  |
| *osmY* | **-2.3** | **-2.6** | -1.1 | (1) |
| **Attachment /Mobility** | | | |  |
| *ydeQ* | **-1.6** | **-1.8** | **-1.5** |  |
| *ydeR* | **-1.5** | **-2.6** | **-1.6** |  |
| *ydeS* | -1.4 | **-2.3** | **-1.7** |  |
| *ydeT* | **-1.4** | **-2.5** | **-1.7** |  |
| *gmr* | **-1.5** | **-1.9** | -1.3 |  |
| *YdaM* | **-1.5** | **-1.8** | -1.2 |  |
| *bhsA* | **-1.9** | **-2.2** | -1.1 |  |
| *crl* | **-1.8** | **-2.0** | -1.0 | (1).(3) |
| **Non-coding RNA** | | | |  |
| *rttR(tpR)* | **-1.8** | -1.4 | 1.0 |  |
| *tpr* | **-1.7** | **-1.4** | -1.0 |  |
| *ryeA* | **-1.5** | **-2.0** | -1.4 |  |
| *IS092* | **-2.0** | **-2.9** | -1.1 |  |
| *ryeE* | **1.8** | **1.7** | 1.1 |  |
| *dsrA* | 1.1 | -1.2 | **-1.7** |  |
| **Lipid metabolism** | | | |  |
| *dacC* | **-1.6** | **-1.7** | -1.3 |  |
| *tadA* | **-1.7** | **-1.7** | -1.1 |  |
| *pgpC* | **-2.2** | **-2.0** | -1.4 |  |
| *eptB* | **-1.4** | **-1.9** | **-1.4** |  |
| **Transcription** | | | |  |
| rsd | -1.4 | **-2.0** | -1.2 | (1).(2) |
| **Translation** | | | |  |
| *rmf* | 1.1 | 1.0 | **-1.7** |  |
| *deaD* | 1.4 | **-1.8** | 1.2 |  |
| **Degradation** | | | |  |
| *clpA* | 1.4 | **-1.8** | 1.1 | (1).(2) |
| **Unknown function** | | | |  |
| *ycgZ* | **-3.4** | **-4.8** | 1.0 | (1).(2) |
| *ymgA* | **-2.8** | **-3.4** | 1.0 | (1).(2) |
| *ariR* | **-4.8** | **-5.9** | 1.0 | (1).(2) |
| *ymgC* | **-4.0** | **-3.8** | 1.0 | (1).(2) |
| *ymgD* | **3.2** | **2.8** | -1.2 | (1) |
| *ymgG* | **3.5** | **3.4** | 1.0 | (1) |
| *yejG* | **-7.7** | **-8.3** | -1.1 | (1).(2) |
| *ylaC* | **-2.8** | **-3.0** | -1.4 | (1).(2) |
| *ybaY* | **-2.2** | **-2.9** | -1.2 | (1).(2) |
| *ybaP* | **-1.9** | **-1.5** | -1.2 |  |
| *yjiR* | **-1.8** | **-1.5** | -1.1 | (1).(2) |
| *rarD* | **-1.7** | **-1.7** | -1.1 | (1) |
| *yigI* | **-3.0** | **-2.7** | 1.1 | (1) |
| *ynaJ* | **-1.6** | **-2.1** | -1.2 | (1).(2) |
| *yjcB* | **-1.6** | **-2.0** | -1.3 |  |
| *yqeF* | **-1.6** | **-2.0** | -1.1 | (1) |
| *csiD* | **-1.7** | -1.3 | -1.3 |  |
| *ybjG* | **-1.5** | **-1.8** | -1.2 |  |

**S2 Table. Genes displaying Fre dependent change in expression in *freΔ68* and Δ*fre*. Results from Fre overproduction are also shown (pFRE).** Gene expression is represented as ratio relative to wt MG1655. Fold changes for genes that are repressed are expressed in negative values. All genes in a given operon where a relevantly expressed gene is located are shown; the gene whose expression is most affected in the operon is highlighted in red (repressed) or green (overexpressed). Genes coding for dehydrogenases are highlighted in yellow. Genes coding for cytochrome terminal oxidase are highlighted in blue. Gene products inhibited by NADH are followed by an asterisk. (1) Operons or genes that are differentially expressed in a Δ*arcA* strain compared to wild-type [27]. (2) Operons or genes directly regulated by ArcA [27]. (3) Operons or genes that are differentially expressed in a Δ*arcA* strain compared to wild-type [26].
