# Supplementary material for: Re-wiring of energy metabolism promotes viability during hyperreplication stress in E. coli
Source: PLoS Genet. 2017 Jan 27;13(1):e1006590. doi: 10.1371/journal.pgen.1006590 (PMC5302844; doi:10.1371/journal.pgen.1006590)
Supplement: S3 Table — The indicated cells were grown in AB minimal medium supplemented with 0.2% glucose and 0.5% casamino acids. Relative abundance of cydA, sdhD and cyoA mRNA relative to rpoA mRNA was measured by quantitative RT-PCR. Shown is the mean ± s.d. (n = 3). For comparison, values for the same genes derived from the microarray analysis are included. (DOCX) [file pgen.1006590.s003.docx]

|  | **wt** | | ***fre*Δ68 ** | | ***iscUC63F*** | |
| --- | --- | --- | --- | --- | --- | --- |
|  | **RT qPCR** | **Microarray** | **RT qPCR** | **Microarray** | **RT qPCR** | **Microarray** |
| *cydA* | **1.0 (0.2)** | **1** | **33.1 (11.1)** | **6.4** | **1.7 (0.5)** | **1.3** |
| *sdhD* | **1.0 (0.1)** | **1** | **-10.5 (6.8)** | **-6.3** | **-6.7 (4.3)** | **-2.3** |
| *cyoA* | **1.0 (0.1)** | **1** | **-7.0 (2.8)** | **-3.2** | **-1.2 (0.1)** | **1.0** |

**S3 Table. Level of *cydA*, *sdhD* and *cyoA* expression measured by Quantitative PCR.** The indicated cells were grown in AB minimal medium supplemented with 0.2% glucose and 0.5% casamino acids. Relative abundance of *cydA*, *sdhD* and *cyoA* mRNA relative to *rpoA* mRNA was measured by quantitative RT-PCR. Shown is the mean ± s.d. (n=3). For comparison, values for the same genes derived from the microarray analysis are included.
