# Supplementary material for: Re-wiring of energy metabolism promotes viability during hyperreplication stress in E. coli
Source: PLoS Genet. 2017 Jan 27;13(1):e1006590. doi: 10.1371/journal.pgen.1006590 (PMC5302844; doi:10.1371/journal.pgen.1006590)
Supplement: S1 Fig — Cells were grown aerobically in AB minimal medium supplemented with 0.2% glucose and 0.5% casamino acids except hda cells that were grown anaerobically and shifted for 4 hours to aerobic conditions. Wild-type cells treated for 30 minutes with 2μg/ml ciprofloxacin are used as strand break control. Top: PFGE gel. Bottom: line scan analysis of relevant lanes. (PDF) [file pgen.1006590.s004.pdf]

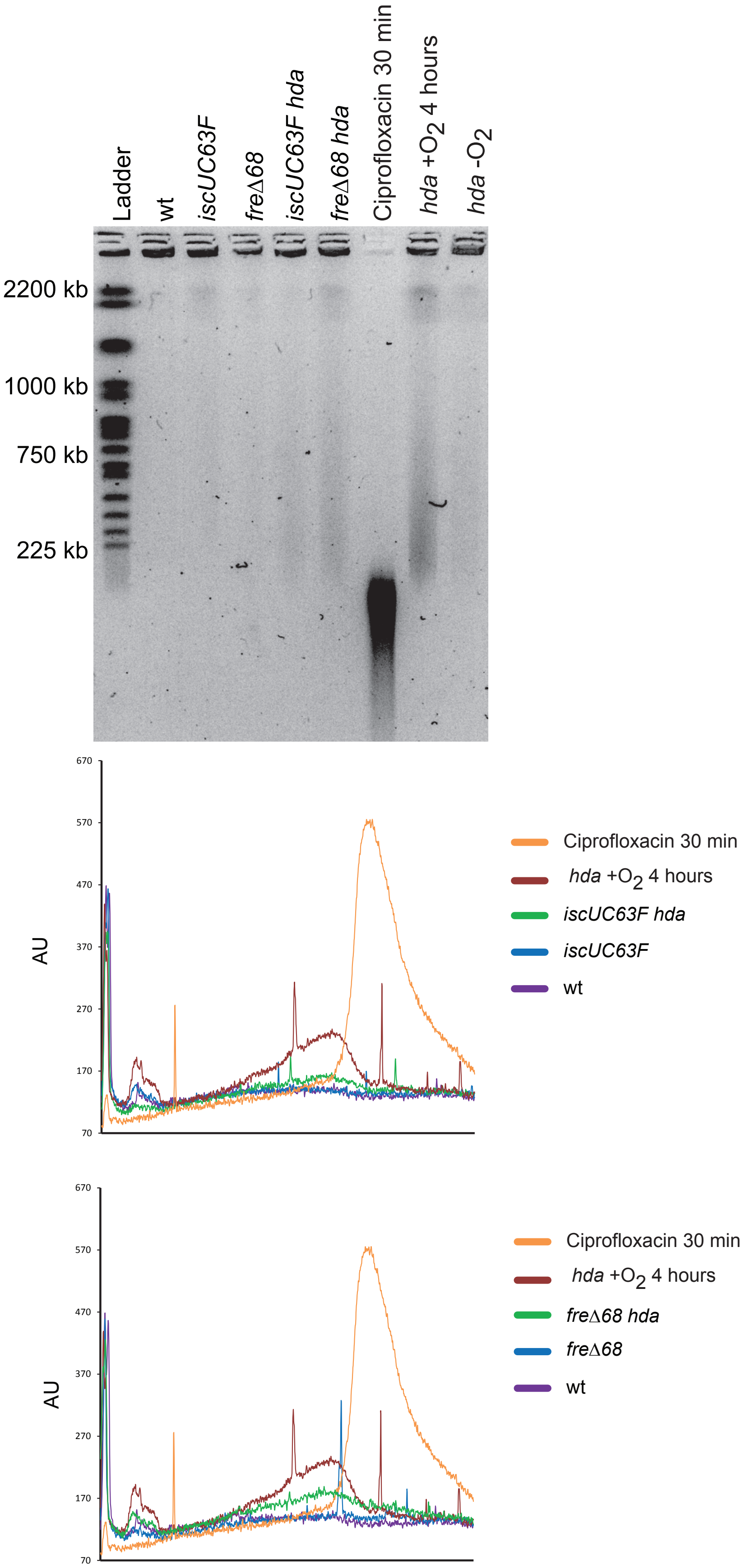

S1 Fig. Vizualization of strand breaks by PFGE. Cells were grown aerobically in AB minimal medium supplemented with 0.2% glucose and 0.5% casamino acids except *hda* cells that were grown anaerobically and shifted for 4 hours to aerobic conditions. Wild-type cells treated for 30 minutes with 2µg/ml ciprofloxacin are used as strand break control. Top: PFGE gel. Bottom :line scan analysis of relevant lanes.
