# Supplementary material for: Re-wiring of energy metabolism promotes viability during hyperreplication stress in E. coli
Source: PLoS Genet. 2017 Jan 27;13(1):e1006590. doi: 10.1371/journal.pgen.1006590 (PMC5302844; doi:10.1371/journal.pgen.1006590)
Supplement: S2 Fig — A) The hda::cat mutation was introduced into wild-type cells under anaerobic conditions, restreaked on AB minimal medium plates supplemented with 0.2% glycerol along with wild-type and hda iscUC63F cells and incubated aerobically. B) The hda::cat mutation was introduced into wild-type cells under anaerobic conditions, restreaked on AB minimal medium plates supplemented with 0.2% glucose and 0.5% casamino acids along with wild-type and hda iscUC63F cells and incubated aerobically. C) Hda clones obtained on AB minimal medium supplemented with 0.2% glycerol were restreaked on LB plates along with wild-type and hda iscUC63F cells and incubated aerobically. D) Cells were grown exponentially in AB minimal medium supplemented with 0.2% glycerol and treated with rifampicin and cephalexin prior to flow cytometric analysis. E) Cells were grown exponentially in AB minimal medium then shifted to AB minimal medium supplemented with 0.2% glucose and 0.5% casamino acids medium for 3 mass doubling time or F) LB medium for 3 mass doubling time and treated with rifampicin and cephalexin prior to flow cytometric analysis. Each panel represents a minimum of 30000 cells. The average ori/cell (O/C), ori/mass (O/M) relative to wild-type and mass doubling time (τ) are inserted in the histograms. (PDF) [file pgen.1006590.s005.pdf]

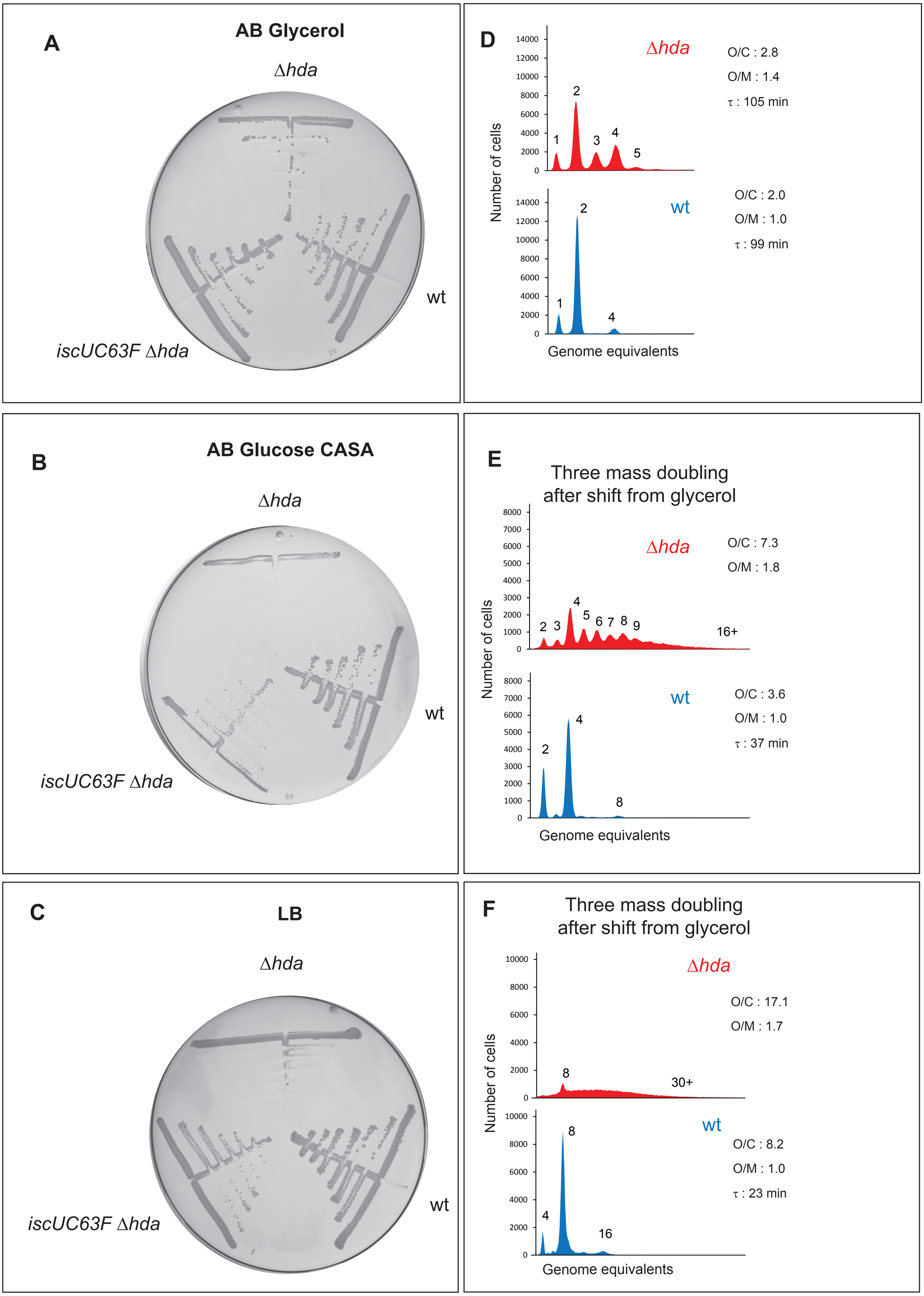
A
AB Glycerol
Δhda
wt
iscUC63F Δhda
D
Δhda
O/C : 2.8
O/M : 1.4
τ : 105 min
wt
O/C : 2.0
O/M : 1.0
τ : 99 min
Number of cells
Genome equivalents
B
AB Glucose CASA
Δhda
wt
iscUC63F Δhda
E
Three mass doubling
after shift from glycerol
Δhda
O/C : 7.3
O/M : 1.8
16+
wt
O/C : 3.6
O/M : 1.0
τ : 37 min
Number of cells
Genome equivalents
C
LB
Δhda
wt
iscUC63F Δhda
F
Three mass doubling
after shift from glycerol
Δhda
O/C : 17.1
O/M : 1.7
30+
wt
O/C : 8.2
O/M : 1.0
τ : 23 min
Number of cells
Genome equivalents

S2 Fig. Hda is dispensable during slow growth. A) The *hda*::*cat* mutation was introduced into wild-type cells under anaerobic conditions, restreaked on AB minimal medium plates supplemented with 0.2% glycerol along with wild-type and *hda iscUC63F* cells and incubated aerobically. B) The *hda*::*cat* mutation was introduced into wild-type cells under anaerobic conditions, restreaked on AB minimal medium plates supplemented with 0.2% glucose and 0.5% casamino acids along with wild-type and *hda iscUC63F* cells and incubated aerobically. C) *Hda* clones obtained on AB minimal medium supplemented with 0.2% glycerol were restreaked on LB plates along with wild-type and *hda iscUC63F* cells and incubated aerobically. D) Cells were grown exponentially in AB minimal medium supplemented with 0.2% glycerol and treated with rifampicin and cephalexin prior to flow cytometric analysis. E) Cells were grown exponentially in AB minimal medium then shifted to AB minimal medium supplemented with 0.2% glucose and 0.5% casamino acids medium for 3 mass doubling time or F) LB medium for 3 mass doubling time and treated with rifampicin and cephalexin prior to flow cytometric analysis. Each panel represents a minimum of 30000 cells. The average *ori*/cell (O/C), *ori*/mass (O/M) relative to wild-type and mass doubling time (τ) are inserted in the histograms.
