# Supplementary material for: Re-wiring of energy metabolism promotes viability during hyperreplication stress in E. coli
Source: PLoS Genet. 2017 Jan 27;13(1):e1006590. doi: 10.1371/journal.pgen.1006590 (PMC5302844; doi:10.1371/journal.pgen.1006590)
Supplement: S3 Fig — Cells were grown exponentially in LB medium and treated with rifampicin and cephalexin prior to flow cytometric analysis. Each panel represents a minimum of 30000 cells. The average ori/cell (O/C), ori/mass (O/M) relative to wild-type and mass doubling time (τ) are inserted in the histograms. (PDF) [file pgen.1006590.s006.pdf]

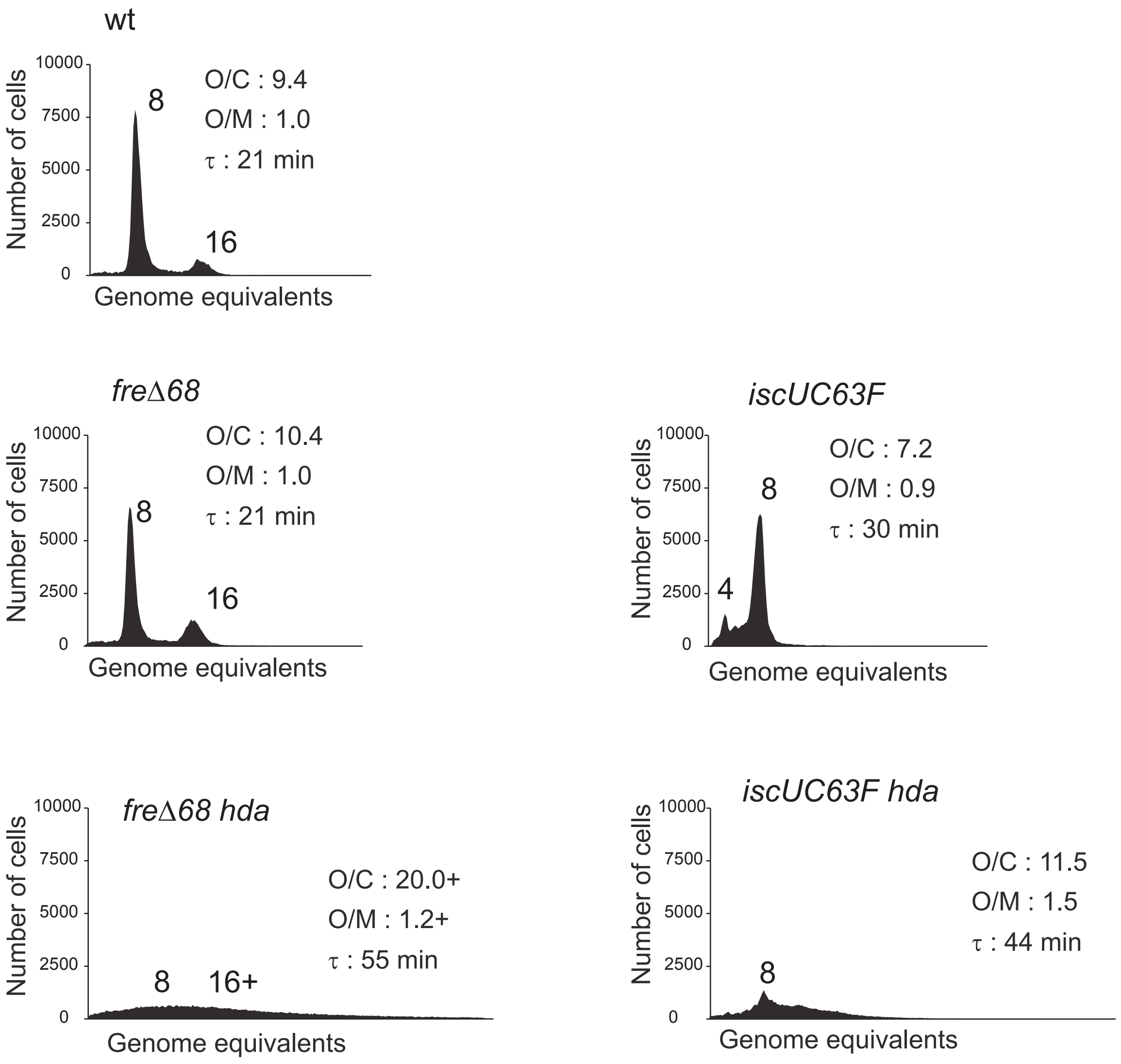

S3 Fig. Cell cycle profile of *iscU* and *fre* mutants grown in LB. Cells were grown exponentially in LB medium and treated with rifampicin and cephalexin prior to flow cytometric analysis. Each panel represents a minimum of 30000 cells. The average ori/cell (O/C), ori/mass (O/M) relative to wt and mass doubling time (τ) are inserted in the histograms.
