# Supplementary material for: Re-wiring of energy metabolism promotes viability during hyperreplication stress in E. coli
Source: PLoS Genet. 2017 Jan 27;13(1):e1006590. doi: 10.1371/journal.pgen.1006590 (PMC5302844; doi:10.1371/journal.pgen.1006590)
Supplement: S4 Fig — The hda::cat mutation was introduced into the indicated strains under anaerobic conditions, restreaked on LB agar and incubated aerobically. (PDF) [file pgen.1006590.s007.pdf]

ΔatpA ΔhdaΔcydB *ΔatpA Δhda*

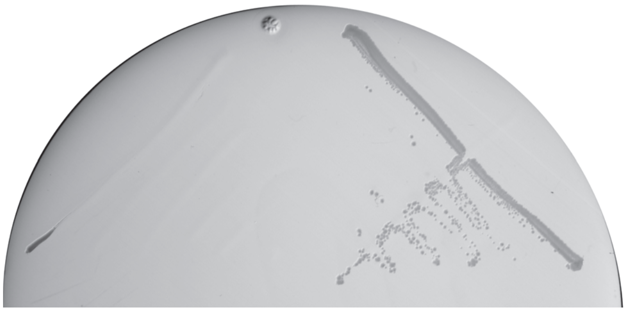

ΔatpB Δhda ΔcydB *ΔatpB Δhda*

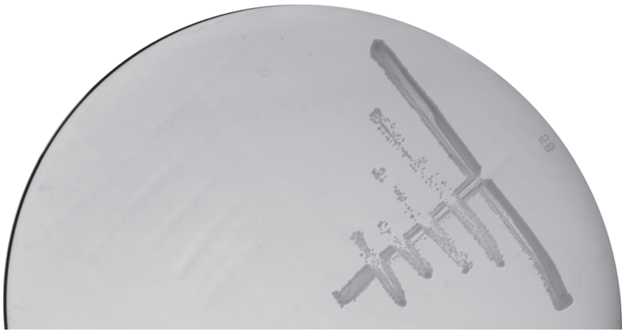

iscUC63F ΔcydB Δhda *iscUC63F Δhda*

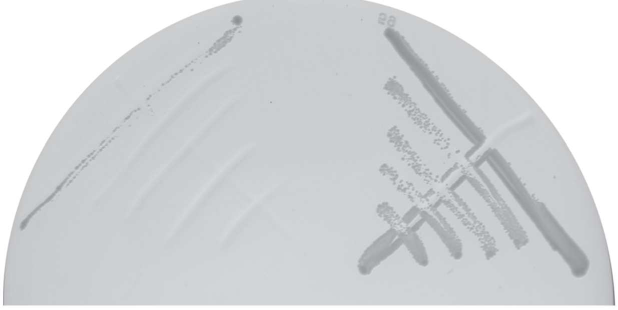

S4 Fig. Cytochrome bd-1 function is required for *atpA*, *atpB* and *iscUC63F* survival in absence of *hda*. The *hda*::*cat* mutation was introduced into the indicated strains under anaerobic conditions, restreaked on LB agar and incubated aerobically.
