# Supplementary material for: Re-wiring of energy metabolism promotes viability during hyperreplication stress in E. coli
Source: PLoS Genet. 2017 Jan 27;13(1):e1006590. doi: 10.1371/journal.pgen.1006590 (PMC5302844; doi:10.1371/journal.pgen.1006590)
Supplement: S5 Fig — The expression level of selected OxyR and SoxRS controlled genes relative to wild-type extracted from the microarray experiment. (PDF) [file pgen.1006590.s008.pdf]

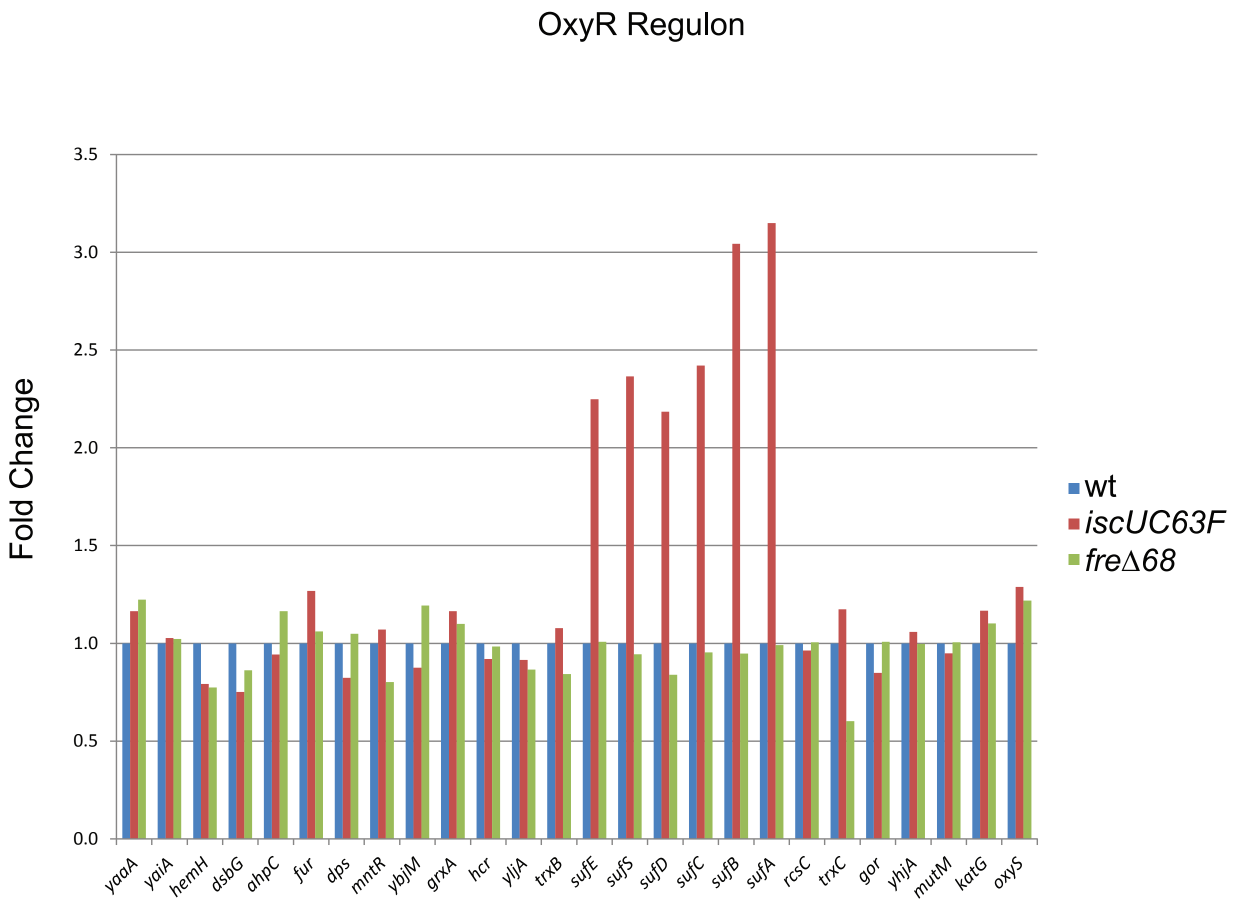

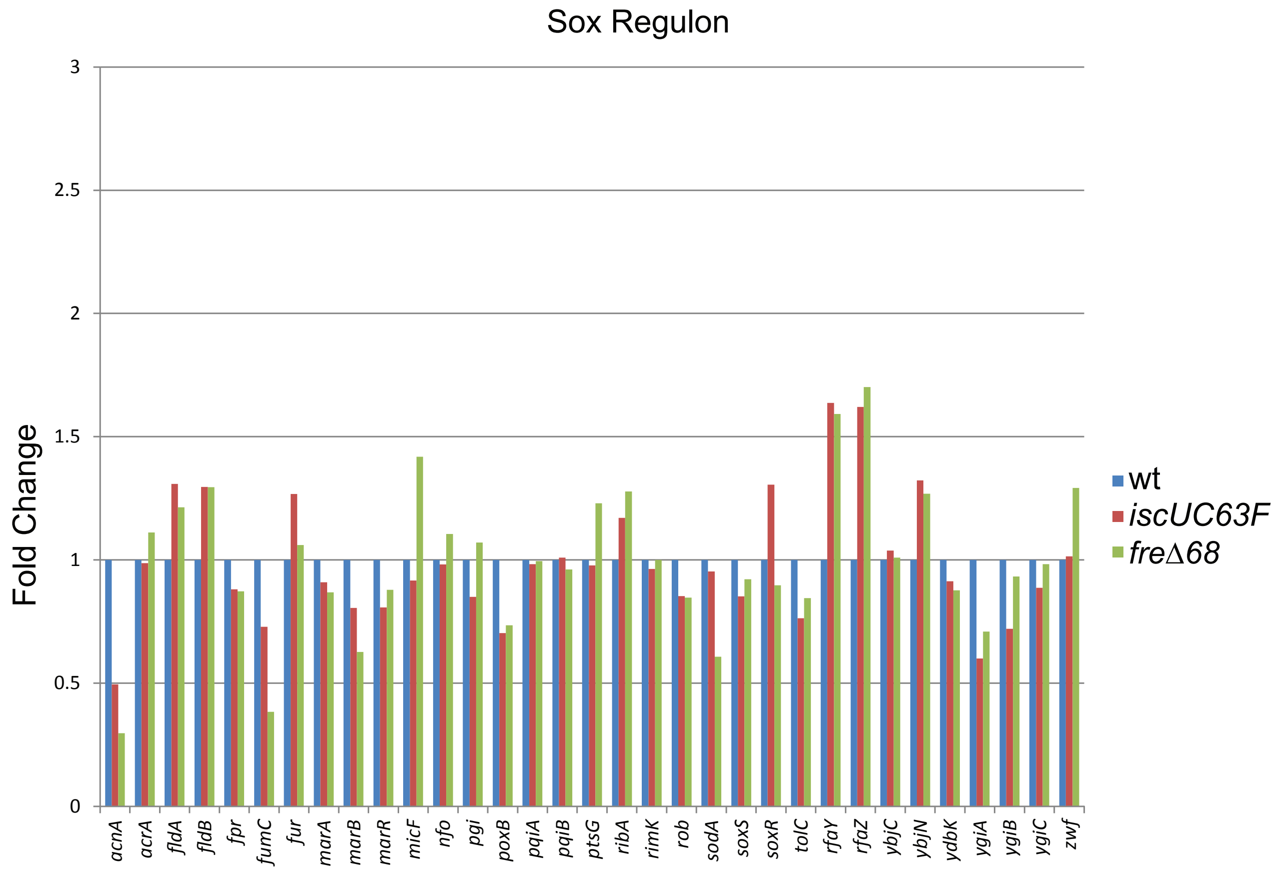

S5 Fig. The OxyR and SoxRS regulons in *iscU* and *fre* mutants. The expression level of selected OxyR and SoxRS controlled genes relative to wild-type extracted from the microarray experiment.
