# Supplementary material for: Re-wiring of energy metabolism promotes viability during hyperreplication stress in E. coli
Source: PLoS Genet. 2017 Jan 27;13(1):e1006590. doi: 10.1371/journal.pgen.1006590 (PMC5302844; doi:10.1371/journal.pgen.1006590)
Supplement: S6 Fig — Expression of katG::lacZ in cells growing in LB medium was measured by β-galactosidase assay. The results are expressed relative to wild-type. (PDF) [file pgen.1006590.s009.pdf]

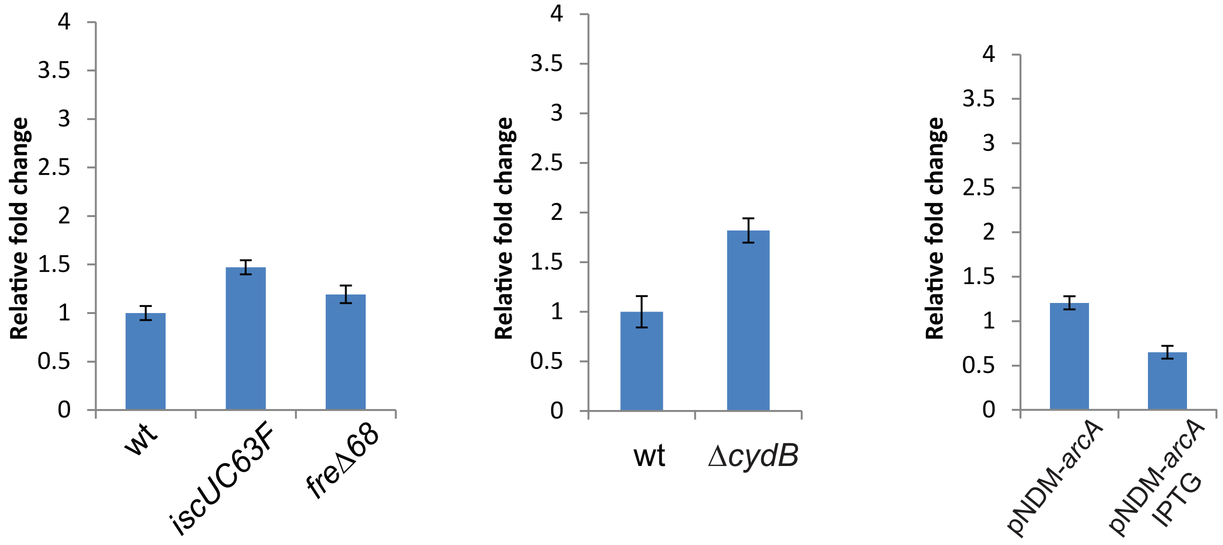


S6 Fig.
*katG* expression in *iscUC63F* and *fre*Δ68, Δ*cydB* and ArcA overproducing strains. Expression of *katG*::*lacZ* in cells growing in LB medium was measured by β-galactosidase assay. The results are expressed relative to wild type.
