# Supplementary material for: Re-wiring of energy metabolism promotes viability during hyperreplication stress in E. coli
Source: PLoS Genet. 2017 Jan 27;13(1):e1006590. doi: 10.1371/journal.pgen.1006590 (PMC5302844; doi:10.1371/journal.pgen.1006590)
Supplement: S7 Fig — The sodA::kan and sodB::kan mutations were introduced into hda freΔ68 cells under anaerobic conditions and restreaked under aerobic conditions on LB agar. (PDF) [file pgen.1006590.s010.pdf]

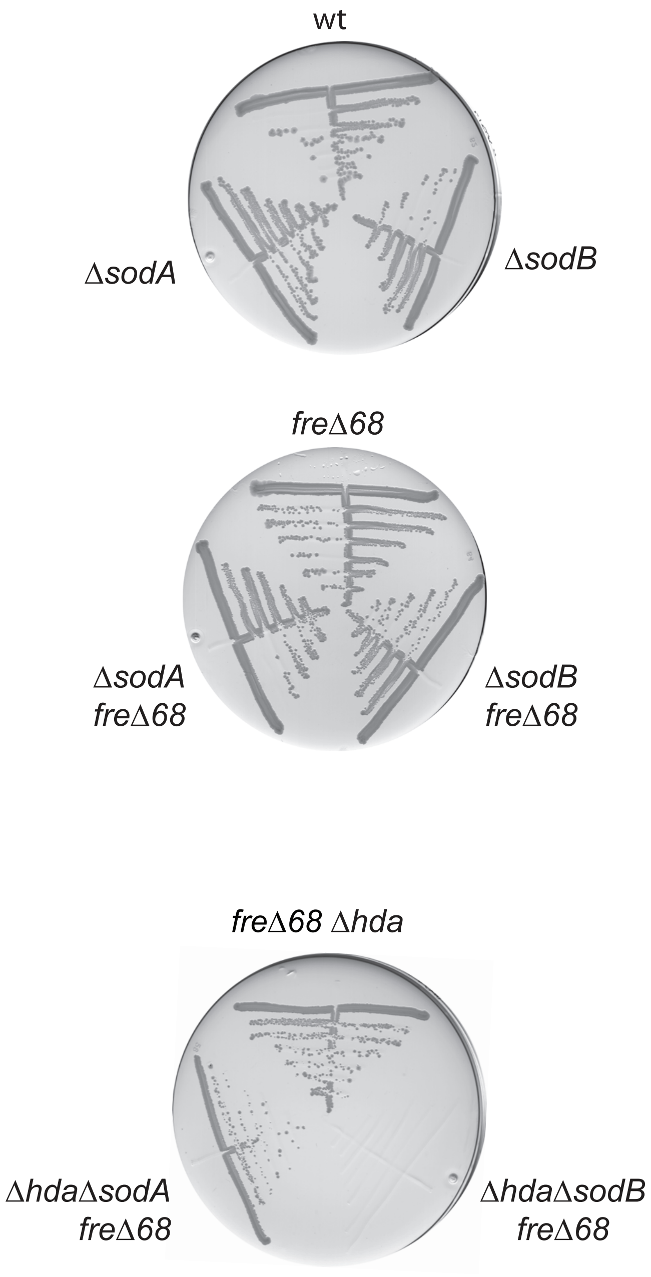

S7 Fig. Effect of *sodA* and *sodB* mutations on growth of *hda fre*Δ*68* cells
The *sodA*::*kan* and *sodB*::*kan* mutations were introduced into *hda fre*Δ*68* cells under anaerobic conditions and restreaked under aerobic conditions on LB agar.
